# Nanoencapsulation of phase change materials for advanced thermal energy storage systems

**DOI:** 10.1039/c8cs00099a

**Published:** 2018-04-16

**Authors:** E. M. Shchukina, M. Graham, Z. Zheng, D. G. Shchukin

**Affiliations:** a Stephenson Institute for Renewable Energy , Department of Chemistry , University of Liverpool , Crown Street , L69 7ZD , Liverpool , UK . Email: d.shchukin@liverpool.ac.uk

## Abstract

Phase change materials (PCMs) allow the storage of large amounts of latent heat during phase transition. They have the potential to both increase the efficiency of renewable energies such as solar power through storage of excess energy, which can be used at times of peak demand; and to reduce overall energy demand through passive thermal regulation. 198.3 million tons of oil equivalent were used in the EU in 2013 for heating. However, bulk PCMs are not suitable for use without prior encapsulation. Encapsulation in a shell material provides benefits such as protection of the PCM from the external environment and increased specific surface area to improve heat transfer. This review highlights techniques for the encapsulation of both organic and inorganic PCMs, paying particular attention to nanoencapsulation (capsules with sizes <1 μm). We also provide insight on future research, which should focus on (i) the development of multifunctional shell materials to improve lifespan and thermal properties and (ii) advanced mass manufacturing techniques for the economically viable production of PCM capsules, making it possible to utilize waste heat in intelligent passive thermal regulation systems, employing controlled, “on demand” energy release/uptake.

## Abstract 2

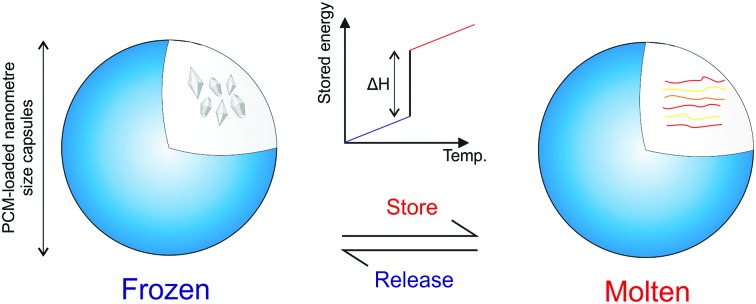
A review focusing on phase change materials for thermal energy storage, particularly their nanoencapsulation, and insight into future research possibilities.

## 1. Introduction

With each passing year, energy becomes more crucial in modern society. A one third increase in demand is predicted by 2035.^1^ Fossil fuels have been humanity's greatest energy resource since the dawn of the Industrial Revolution. In 2001, global consumption of energy was 4.25 × 10^20^ J, of which 86% was produced by fossil fuels.^2^ However, oil, coal and natural gas reserves are not infinite, and have had an enormous impact on our environment. Large amounts of emitted greenhouse gases in the atmosphere have led to relatively rapid climate change and acidification of the oceans in the last 250 years. Environmental change can already be observed by the behaviour of wild plants and animals, with their geographical distribution being affected.^3^,^4^ Fossil fuels can also result in major political problems. Their uneven distribution can cause interdependencies between countries and may even lead to conflict.^5^

Therefore, it is important to develop cleaner energy sources. The best possible energy sources are renewable energies. They are unlimited in the amount of energy they can supply, and often produce zero greenhouse gases. Unfortunately, due to the high demand for immediate power, renewable energies are not currently reliable or economically viable enough to fully replace oil, coal and natural gas. It is vital to develop energy storage systems to ensure clean energy can be provided round the clock.

Solar power is considered the most promising renewable energy due to its abundance, zero cost and lack of emissions.^6^ The US Department of Energy calculated that the worldwide consumption of energy in 2001 could be met with less than 1 and a half hours of sunlight.^7^,^8^

The major drawback of renewable energies such as solar power is their intermittency – when the sun is not shining, no energy can be produced. This is where thermal energy storage is of great importance. Excess of thermal energy can be stored using an energy storage media, which acts as energy sink. The energy can then be released during peak hours to meet demand, known as peak shifting. Factors involved in the selection of heat storage materials include cost, storage density and reliability.

### Thermal energy storage approaches

**Fig. 1:**
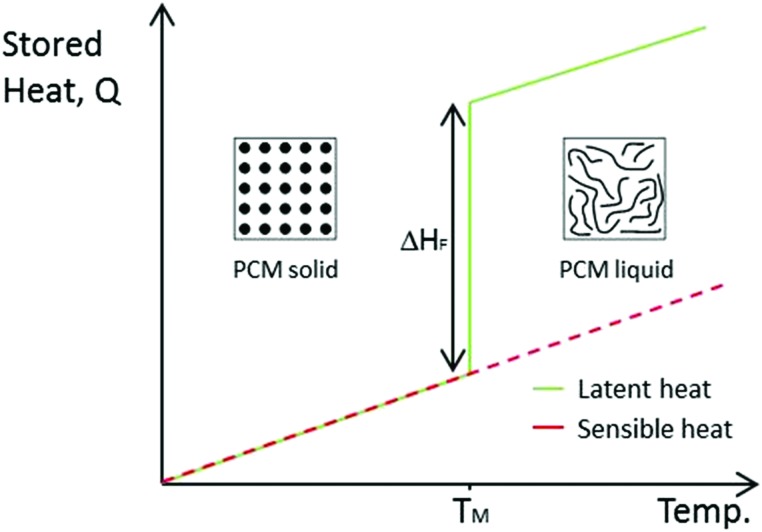
Comparison between SHS and LHS, Δ*H*_F_ is the latent heat of fusion during melting. *T*_M_ is the melting temperature.

There are three main approaches for thermal energy storage: sensible heat storage (SHS), latent heat storage (LHS) and thermochemical energy storage (TCS). Sensible heat refers to heat that can be detected (“sensed”) by a temperature change in a linear relationship with temperature (as seen in Fig. 1). The heat stored is dependent on the specific heat capacity of the material. SHS is the simplest and most developed form of heat storage, however, it suffers from low energy density and loss of thermal energy at any temperature.^9^

Latent heat storage refers to heat transfer associated with phase transitions, which cannot be detected with a thermometer. LHS is more efficient and has a far superior storage density than SHS.

Materials that utilise LHS are known as phase change materials (PCMs). Examples of phase transitions include melting and freezing (solid–liquid), evaporation and condensation (liquid–gas) or changes in crystalline structure (solid–solid). Essentially, the energy associated with these changes corresponds to the number of chemical bonds broken. Therefore, solid–gas transitions store the highest amount of energy. However, the large volume change of these transitions means pressurised containers are required. Solid–solid and solid–liquid PCMs have been researched since the oil crisis of the 1970s brought energy to the fore of scientific research. However, once the crisis was down, they were largely forgotten until the 2000s. With the current focus on clean energy sources, PCMs have become widely studied at an increasing rate. As can be seen from Fig. 1, an ideal LHS material stores a large amount of heat isothermally during melting. Once the material freezes, this energy is released. PCMs also store thermal energy sensibly whilst not undergoing phase transition. PCMs are far more efficient than SHS materials, especially over the small temperature range associated with their phase transition.

In 1983, Abhat^10^ outlined the ideal properties for a PCM:

1. Thermodynamic:

• Melting temperature (*T*_M_) in desired application range.

• High latent heat of fusion.

• High density.

• High specific heat for additional SHS.

• High thermal conductivity.

• Congruent melting.

• Small volume changes during phase transition.

• No supercooling.

2. Chemical:

• Chemically stable over long periods.

• Non-corrosive to container materials.

• Non-flammable, non-toxic and non-explosive.

3. Economic:

• Low cost.

• Available in large quantities.

However, there are no PCMs to date which fit all these criteria.

Thermochemical energy storage gives the highest energy density of all, around 5 to 10 times greater than LHS and SHS, respectively. TCS relates to energy stored and released during controlled reversible chemical reactions. Despite the progress made with TCS and its potential for high temperature applications, it has considerable issues with long-term stability. Reactions must have constant conversion efficiency without degradation of energy storage capacity over long periods of time.^11^ Essentially, the major problem for TCS is the lack of research and understanding. Currently, SHS has been developed to an industrial level, LHS to pilot plant scale, while TCS has only been tested on a laboratory scale.^11^ There is a recent review focused on TCS materials which we recommend to readers.^12^ TCS may be valuable in future, but LHS should be the focus for immediate research to solve energy storage issues.

PCMs have several applications, which can be grouped into two categories – thermal regulation and thermal energy storage. Thermal regulation is highly important, and PCMs with a *T*_M_ in the desired application range can prevent temperature fluctuations with no energy input. To illustrate the vast potential of passive thermal regulation, PCMs with *T*_M_s in the human comfort range (10–25 °C) can be used to air condition buildings. Buildings account for approximately 40% of global energy usage, a large amount used for air conditioning.^13^–^15^ In 2013, 198.3 million tons oil equivalent were used for space heating in the EU alone. This is especially true of modern lightweight constructions, which suffer from large temperature swings. Other applications benefitting from thermal regulation include Li batteries,^16^–^18^ photovoltaics,^19^–^21^ spacecraft/spacesuits^22^ and textiles.^23^–^25^

PCMs can also be used as energy storage media for waste heat from industrial processes^26^ or fuel cells.^27^ The waste heat can then be reused, for example, it can be transported to off-site purchasers for applications such as greenhousing.^28^ Energy storage can also be employed to improve the efficiency of concentrated solar power, using high temperature PCMs with transition temperatures above 300 °C (salts or metals).^29^ Few attempts have been made to encapsulate high temperature PCMs on the macro^30^,^31^ and micro^32^ scale. This review (i) surveys the main encapsulation approaches for nanostructured and multifunctional capsule shells and (ii) focuses on the nanoencapsulation of low temperature paraffin waxes and salt hydrate. For a more detailed PCM loading into matrix-type cores or organic shells, we strongly recommend two recent papers.^33^,^34^

### PCM classification

**Fig. 2:**
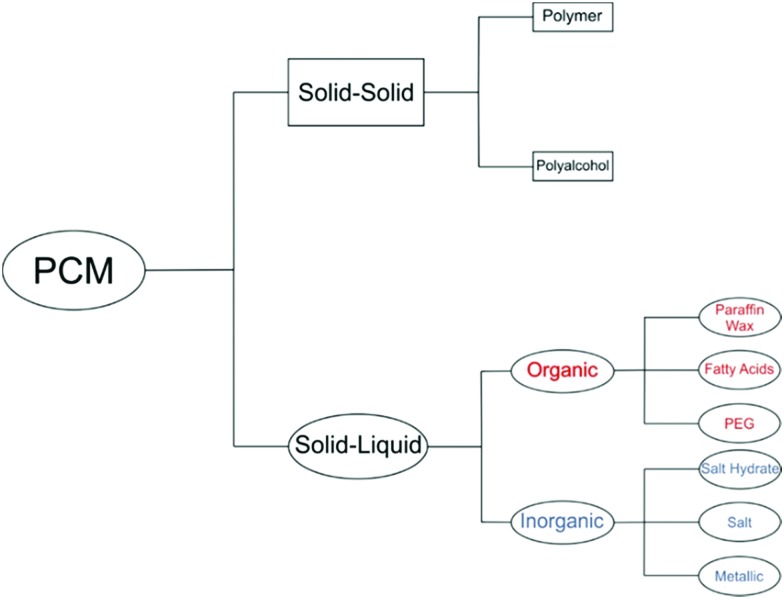
Classification of various solid–solid and solid–liquid PCMs.

PCMs can be classified according to the specific phase transitions they undergo. As mentioned above, the sublimation and evaporation give the highest latent heat of fusion but are not practical due to the large volume change and need for specialised containment to prevent material loss. There are several categories of PCMs, as seen in Fig. 2. Solid–solid PCMs have low latent heat of fusion, and are not considered useful for practical applications. Solid–liquid PCMs give a good balance between high latent heat of fusion and manageable volume change.

Solid–liquid PCMs can be divided into organic or inorganic materials (Fig. 2). Organic PCMs include paraffin wax, fatty acids and polyethylene glycol (PEG), whilst inorganic PCMs can be salt hydrates, salts or metallic.

Paraffin waxes are linear alkanes containing between 8–40 carbon atoms. Paraffins often display additional LHS in the form of solid–solid transitions associated with different crystalline phases. Their disadvantages include low thermal conductivity, bad odour, flammability and high cost.

Paraffin waxes are also non-renewable, as they are refined from petroleum with bleaching agents. Commercial paraffin contains formaldehyde and vinyl chloride as well as benzene, toluene, naphthalene and methyl ethyl ketone which are volatile and carcinogenic in nature, so care must be taken while using this materials in building applications.^35^

Fatty acids can be produced from vegetable based oils which are non-toxic. They have lower flash points and longer flame propagation than paraffins. However, their high cost (even higher than paraffin waxes) has rendered them unusable in practical applications. Due to the large volume change on melting, they must also be contained.

Salt hydrates (also known as crystallohydrates) are the major class of inorganic PCMs, and most promising PCMs overall due to their high latent heat, high energy storage density, low cost, abundance, reasonable thermal conductivity and wide variety of melting temperatures in the domestic application range (5–130 °C). Their favourable properties compared to paraffin waxes are displayed in Table 1. They have the general formula M·*n*H_2_O where M is a metal salt and *n* is the hydration number. Salt hydrates’ high energy density is especially attractive as less material is required, reducing the necessary volume of containers and reducing costs further. Salt hydrates have specific densities in the region of 1500–2000 kg m^–3^, whereas paraffin waxes have specific densities of around 900 kg m^–3^. Combined with their higher latent heats of around 200–250 J g^–1^ compared with 150–200 J g^–1^ for paraffins,^36^ their energy storage ability is far greater. Paraffin waxes have similar latent heat values to some salt hydrates. However, when the latent heat per unit volume is quoted, salt hydrates will demonstrate greater energy storage ability. Salt hydrates have energy densities of around 250–400 J dm^–3^ compared with around 125–200 J dm^–3^ for paraffin waxes.^10^,^37^,^38^ Data for energy density in J dm^–3^ is sparse in the literature, but is very useful when considering sizes of the macroscale thermal energy storage unit.

**Table 1:** Comparison of key properties of paraffin wax and salt hydrate PCMs. Data taken from Zalba *et al.*^36^ and Abhat^10^

	Paraffin wax	Salt hydrate
Energy density	125–200 J dm^–3^	250–400 J dm^–3^
Latent heat	150–200 J g^–1^	150–250 J g^–1^
*T*_M_ range	–60 to 80 °C	5–130 °C
Thermal conductivity (solid phase)	0.2 W m^–1^ K^–1^	0.7–1 W m^–1^ K^–1^
Supercooling	No	Yes
Congruent melting	Yes	No

An advantageous property of salt hydrates that is not fully realised is the formation of mixtures and eutectics. When salt hydrates are mixed, their *T*_M_ is lowered due to the inhibition of crystallisation of the components.^39^ The ratio which results in the lowest possible *T*_M_, always lower than both of the component compounds, is known as a crystallohydrate eutectic. Eutectics have a high latent heat due to the formation of a single phase, and often have reversible phase change without phase separation. Eutectic salts have relatively low thermal conductivity, the same as for single crystallohydrates, which reduces heat transfer.^40^ Research into eutectics is particularly useful as there are a few known pure crystallohydrates with a *T*_M_ in the optimal range for applications such as air conditioning.^41^

Salt hydrates have several disadvantages. Incongruent melting is incomplete melting of the salt hydrate, leading to the irreversible formation of a salt of lower hydration number. This salt then precipitates at the bottom of the melt due to density difference, known as a phase separation. These effects reduce Δ*H* at the desired *T*_M_, and will eventually lead to zero latent heat, rendering salt hydrates chemically unstable, often after very few melting/freezing cycles. Supercooling (calculated from the difference between *T*_M_ and freezing temperature (*T*_F_)) is also a major problem. It is a phenomenon where a material must be cooled far below its freezing point in order to freeze, and is caused by poor heat transfer. This can be as much as 40 °C. Salt hydrates also display corrosiveness towards container materials.^42^,^43^

Salt hydrates have varying levels of toxicity. They are generally non-toxic in nature but they can cause skin or eye irritation and respiratory problems. Most crystallohydrate salts have low prices (as cheap as 100 USD per ton for sodium sulphate decahydrate).^41^

Other major classes of inorganic PCMs are salts and metals, which are most suitable for high temperature applications. They have the widest range of melting temperatures, salts from –86 °C for a 24.8 wt% HCl and water eutectic mix, up to 500+ °C.^44^ Several metals and alloys have *T*_M_s lower than 100 °C; others have *T*_M_s of 1000+ °C. The major advantage of metallic PCMs is their high thermal conductivity,^45^ but they have low storage density. The high mass of metals must also be considered for any practical applications such as their use in building materials, and makes them unsuitable for transportation of heat energy.^23^ Low temperature metallic PCMs such as gallium (*T*_M_ = 29.8 °C) have been used to cool computer chips and USB memory drives.^46^,^47^ It is anticipated in the future that encapsulation techniques for high temperature PCMs will be developed which will make them available for storage of high temperature heat.^29^

Low thermal conductivity of commonly used PCMs is the biggest technological problem facing PCMs now.^48^ This leads to poor life stability of PCM containers and heat exchanger tubing, and also decrease the number of effective cycles they can undergo without any deterioration in their properties.^49^

### Improvement of PCM performance by encapsulation

The practical use of PCMs is hindered by their limitations. For instance, the solid–liquid transition must be suitably contained to prevent leakage. No known PCM fulfils all of their ideal criteria (outlined by Abhat and listed above).^10^ There are two approaches to modify pure PCMs in order to improve their stability and performance. The first one is to make form-stable PCMs, which is a network on micro or macroscale where PCMs are trapped. These networks are open to the local environment and cannot prevent material exchange, which is particularly important for crystallohydrate PCMs to avoid water loss. This type of PCM control has been covered in a comprehensive review by Kenisarin *et al.*^50^

The second is the encapsulation of PCMs into micro and nanocapsules possessing ‘smart shell’ properties: controlled thermal energy release, prevention of material exchange with environment, protection against degradation during heat uptake/release cycles, increased PCM surface area and heat conductivity and the possibility to use the capsules in powder or paste form as additives to convenient materials (concrete, foam, paint, *etc.*) to attain thermal energy storage/release properties. The aim of our review is to highlight the advances in this second approach – to involve readers in the interesting and rapidly expanding area of nanoencapsulation of energy-enriched materials.

## 2. Overview of encapsulation approaches

Capsules can be macro- (>1000 μm), micro- (1–1000 μm) or nanosized (1–1000 nm). Smaller capsules greatly increase the surface-area-to-volume-ratio of the material, which improves heat transfer. For example, it has been predicted that encapsulating PCMs in capsules of 1 mm in size would increase the surface area by 300 m^2^ m^–3^ when compared with the bulk PCM.^1^ Reducing their diameter to the nanometre range would vastly enhance this effect. Other advantages of encapsulating PCMs include prevention of both leakage and reactions with the external environment, corrosion protection for container materials, control over volume change upon melting and improved thermal cycling stability.^51^,^52^ All these properties are crucial to PCM usage in practical applications, so encapsulation can almost be thought of as a “one size fits all” solution. The shell is usually made up of a polymer, as they give a good balance between strength and flexibility. Inorganic shells can also be used, which have higher thermal conductivity (silica is a good example of an inorganic shell for PCMs due to its enhanced heat transfer) but are more brittle. It is also possible to form a composite polymer/inorganic shell combining advantages of each.

To create capsules containing active energy materials, an emulsion of the desired droplet size must first be formed, followed by the formation of shell at the emulsion droplet interface. However, the future level of the encapsulation development requires not only the fabrication of the emulsion systems, but also their functionalization in order to realise multifunctional properties.^53^

### Emulsions

An emulsion is a liquid dispersed in another liquid in which it is not soluble or miscible. This is achieved with surface active agents, widely known as surfactants. Due to their amphiphilic nature, they spontaneously form an initial shell around the dispersed liquid to create droplets known as micelles. Emulsions can be oil-in-water (O/W) or water-in-oil (W/O), depending on the PCM to be encapsulated. Usually, the liquid of least volume is dispersed within the other liquid. W/O emulsions (used for crystallohydrate encapsulation) require careful selection of the continuous oil phase and surfactants to give the highest solubilisation capacity for the dispersed phase.^54^,^55^ The shell material can either polymerise around the droplets, or can be premade and deposited.

It is possible to reduce emulsion droplet size by an external energy input. Common energy inputs include homogenisation and sonication. An emulsion with nanosized droplets formed with a high energy input is known as a miniemulsion (or nanoemulsion). This is in contrast with microemulsions, which also have nanometre sized droplets, but form spontaneously. Miniemulsions are kinetically stable while microemulsions are thermodynamically stable.^56^ Microemulsions require a larger amount of surfactant than miniemulsions, usually at least 20 wt% of surfactants in the oil phase, whereas miniemulsions require 3–10 wt%.^57^ Regular emulsions and miniemulsions are thermodynamically unstable due to the spontaneous minimisation of interfacial area between the two immiscible phases.^58^ Microemulsions, in contrast, display thermodynamic stability as the large amount of surfactants overcome the interfacial energy.^52^ A comparison of the basic features of the emulsions types is displayed in Table 2.

**Table 2:** Comparison of different types of emulsion, taken from Rao *et al.*^59^

Characteristics	Emulsion	Miniemulsion	Microemulsion
Thermodynamic stability	No	No	Yes
Stability lifetime	Seconds to months	Hours to months	Infinite
Droplet size range	1–10 μm	20–200 nm	10–100 nm
Polydispersity	Low	Very low	Very low
Typical particle size	1+ μm	100–300 nm	30–100 nm

Regular homogenisation (such as Ultraturrax) does not provide the required amount of energy to form a miniemulsion, as much energy is lost as heat due to friction.^58^ Regular homogenisation is therefore only effective at producing microcapsules >1 μm in size.

**Fig. 3:**
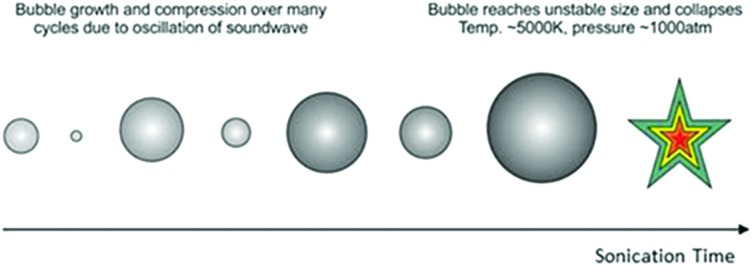
Growth and collapse of bubbles, arising from acoustic cavitation. Once the bubble reaches an unstable size it collapses, giving enormous local temperatures and pressures.

The use of ultrasound for a wide variety of applications is a recent development in the field of materials chemistry. Due to the reverse piezoelectric effect, electrical energy can be converted to mechanical energy using an ultrasonic transducer.^60^ Ultrasound is an advanced physico-chemical process, and has been used for applications including the removal of contaminants from water,^61^ driving reactions,^62^ materials synthesis,^63^ cleaning,^64^ creating new surfaces^65^ and breaking up aggregates of particles.^65^ Ultrasound is defined as sound at frequencies above 16 kHz, which is generally inaudible to adult humans, and creates a huge amount of energy produced through a process known as acoustic cavitation. Although not fully understood, the phenomenon is caused by microscopic bubbles forming and rapidly collapsing, producing localised temperatures above 5000 K and pressures of several thousand bars.^66^,^67^ This process is schematically shown in Fig. 3.

There are three major effects from acoustic cavitation: (i) primary sonochemistry – gas phase chemistry occurring inside the bubbles, (ii) secondary sonochemistry – solution phase chemistry occurring outside the bubbles and (iii) physical effects associated with bubble collapse – a shockwave causing strong turbulent effects such as interparticle collisions.^67^

Miniemulsions are an excellent precursor to the formation of nanocapsules, and sonication is highly efficient at reducing droplet size.^68^ All sonochemical applications require the optimisation of reaction conditions, such as the total time of sonicating and amplitude. Selecting a suitable probe will prevent splashing or foaming of the liquid, effects which lead to a reduction in the power delivered to the solution. A study was undertaken by Asakura *et al.*^69^ to determine how sonochemical efficiency was affected by the amount of liquid in the reactor. They found the optimum liquid height in the reactor is approximately 15 times the height of the wavelength, with the ideal frequency being 200–600 kHz. Lower frequencies mean too few bubbles for cavitation are formed, whereas higher frequencies result in many bubble collisions,^70^ leading to a reduction in internal bubble temperature and therefore energy.^71^ Ultrasonic probes are currently not suitable for industrial use, but have the potential to be scaled up in future, as long as several factors of practicality and safety are taken into account.

Additionally, sonication can initiate polymerisation reactions.^72^ Teo *et al.* polymerised various methacrylate monomers using pulsed sonication to drive the reaction.^62^ Capsule formation could theoretically be designed so that sonication facilitates both miniemulsion and shell formation. As a downside, if high molecular weight polymers are desired, sonication may not be suitable as initiator. Longer polymer chains can be degraded by energy from acoustic cavitation.^65^

### Formation of the stable capsule shell

Capsules have found many uses in applications such as in food technology,^73^,^74^ dyes,^75^ catalysis,^76^,^77^ corrosion inhibition and self-healing^78^,^79^ and drug delivery.^80^–^84^ Their main purpose is to provide protection for the core material and control material and energy exchange between core and external environment. Encapsulation is found throughout nature, for example egg shells and cell membranes. Synthesised capsules can be used to encapsulate many species, including drugs, enzymes,^85^ PCMs and DNA.^86^,^87^ They can also be used as a reaction medium, for example Kang *et al.* showed the rate of a Diels–Alder reaction could be rapidly sped up due to the vastly increased concentration of reactants inside the capsule.^88^ With technological advances over the last half century, preparing capsules with diameters from 1000 μm down to around 40 nm has become possible. Searching the literature using Web of Science, the first mention of microcapsules is from 1964 where Chang reported the encapsulation of enzymes.^89^ Nanocapsules are referenced from 1976 onwards, with an early example being Couvreur *et al.*, who demonstrated how polyacrylamide capsules 200 nm in diameter could encapsulate fluorescein.^90^

Capsule size can play a role in functionality. For instance, in drug delivery nanosized drug-loaded capsules have advantages over regular drugs because certain membranes in the body only allow diffusion of molecules less than 100 nm,^91^ and specific locations in the body can be targeted by specific particles.^92^–^94^ The interest in extremely small capsules is essentially an attempt to increase the surface-area-to-volume ratio (SA/V) of the active material. Increasing SA/V is the driving force for all nanotechnology and is inspired by natural structures such as the alveoli in the lungs. Along with increased surface area, nanocapsules provide increased structural stability compared to microcapsules, which may break whilst being pumped round a heating system.^52^,^95^,^96^

There have been many techniques reported for creating micro- and nanocapsules. These include spray-drying,^97^,^98^ miniemulsion polymerisation,^99^,^100^ precipitation of pre-formed polymers,^101^ layer-by-layer assembly (LbL),^78^,^85^ or other more advanced polymerisation reactions such as radical addition–fragmentation chain-transfer (RAFT)^102^ and the creation of dendrimers.^103^ Each of these techniques has their own advantages and disadvantages. While the deposition of pre-formed materials is a simple process, polymerisation reactions are generally more adaptable.^59^

However, future development of energy capsules should not only concentrate on the fabrication of a single shell around emulsion drops, but also shell functionalization to impart multifunctional properties.^53^ One of the prospective approaches to attain additional functionality to the emulsion particles is the use of the layer-by-layer shell assembly on their surface. This technique permits the step-wise adsorption of various components (polyelectrolytes, nanoparticles, proteins, enzymes, *etc.*) and allows the formation of multilayer shells with nanometre precision.^104^ Capsules with LbL assembled polyelectrolyte shell are used for encapsulation and release of drugs, DNA, dendrimers and enzymes.^105^,^106^

**Fig. 4:**
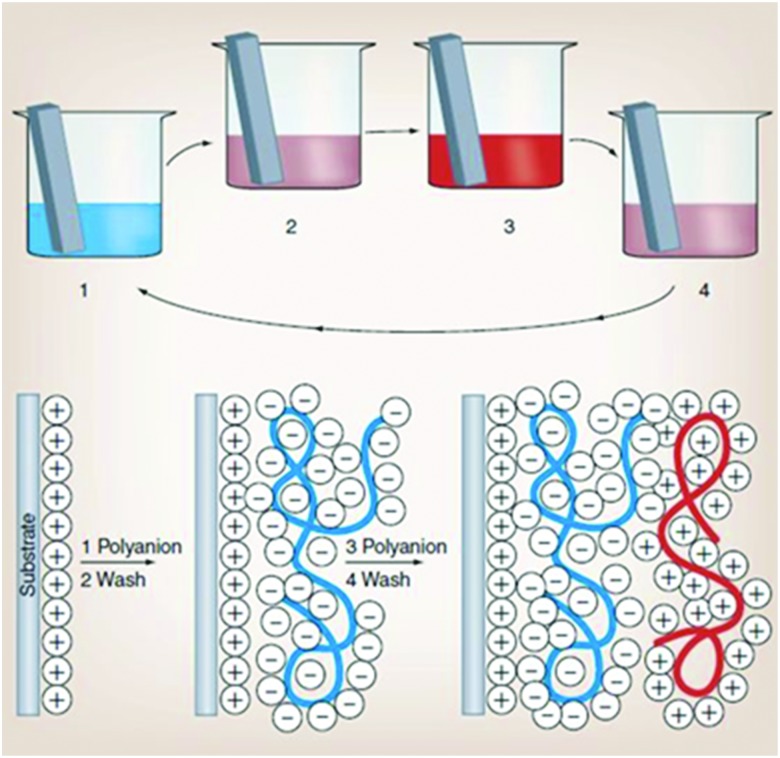
Schematic representation of electrostatically driven layer-by-layer deposition of polyelectrolytes. Reproduced from ref. 104 with permission from the American Association for the Advancement of Science, copyright 1997.

This technique is very simple and based on the iterative adsorption of oppositely charged molecules or nanoparticles on a flat surface or template particle (Fig. 4). A charged surface is immersed into a solution of an oppositely charged polyelectrolyte, followed by a washing step to remove excess polyelectrolyte. This procedure results in changes of the surface charge.

In the next step, the substrate is dipped in a solution of a second, oppositely charged polyelectrolyte. This second polyelectrolyte adsorbs onto the first layer reversing the surface charge. This process can be repeated as many times as desired, yielding multilayered films. In most cases, the technique employs electrostatic forces between oppositely charged polymers and surfaces.^107^ However, other mechanisms can be employed: hydrogen bonding,^108^ covalent bonding,^109^ base-pair interactions,^110^ guest–host interactions,^111^ hydrophobic interactions^112^ or biological recognition.^113^ The use of the LbL technique to prepare capsule shells offers many attractive possibilities. The method allows control over the composition and thickness of the multilayers in the capsule shell. The reduction of the LbL capsule size from microns to nanometers leads to their higher mechanic stability under deformation forces.^114^

**Fig. 5:**
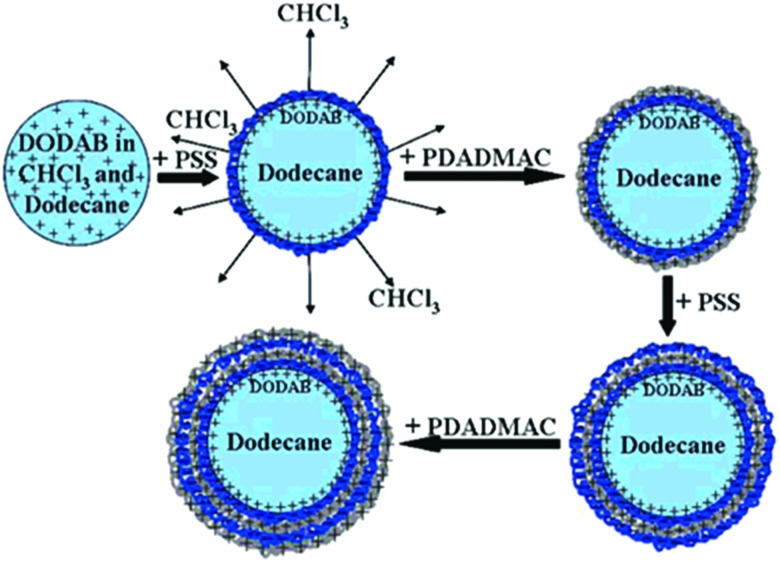
Schematic representation of several steps during LbL polyelectrolyte emulsion encapsulation. Reproduced from ref. 117 with permission from the American Chemical Society, copyright 2008.

Several groups employed LbL technology to encapsulate stable oil-in-water emulsions with a high monodispersity.^115^,^116^ A usual preparation method for LbL coated emulsion carriers involves several steps (Fig. 5).^117^ To stabilize the dispersed oil phase (dodecane) of the initial emulsion, it was doped with a small amount of the cationic surfactant dioctadecyldimethylammonium bromide (DODAB). The colloidal stability of initial emulsion was achieved due to concentrated monolayer of strongly positively charged DODAB (*z*-potential was about +90 mV) at the surface of each droplet.

Then, the subsequent LbL deposition was performed from concentrated aqueous salt-free solutions of polyelectrolytes:poly(styrene sulfonate), PSS, and poly(diallyldimethyl ammonium chloride), PDADMAC. The further repetition of the alternating adsorption steps leads to the formation of oil-containing capsules with desired shell thickness depending on the particular final demand.

The main drawback of the polymer shell for PCM encapsulation is its low thermal conductivity. The LbL approach allows the addition of inorganic nanoparticles as one or several layers of the multilayer shell. A small amount of particles can lead to an enormous increase in thermal conductivity.^118^,^119^

The ultimate level of the capsule shell formation from nanoparticles is called Pickering emulsion (or colloidosomes). Colloidosomes are capsules with shell of nanoparticles localized at the oil–water interface. An economically attractive aspect is the simplicity of the fabrication procedure of such particle stabilized capsules. In principle, only the components (particles, oil, and water) need to be mixed and the application of high shear forces generates capsules with adjustable size. In comparison to surfactant based capsule production, no subsequent purification is required if precise process parameters are met, as all solids will self-assemble on the available oil–water interface. The application of the LbL assembly approach for colloidsomes will close the interstitial pores of the nanoparticulated shell thus preventing material exchange between capsule core and local environment.

**Fig. 6:**
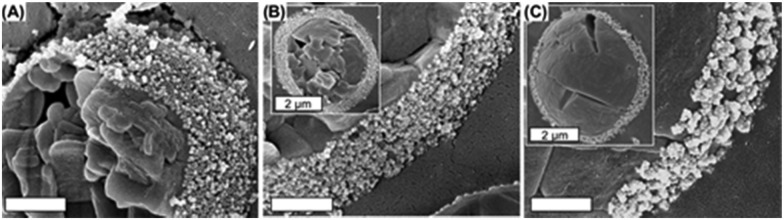
Cryo SEM images of dodecane droplets stabilized with silica–poly(allylamine hydrochloride) particles. Corresponding pH values of emulsions are (a) 8.5, (b) 9.1, and (c) 9.8. Scale bar equals 500 nm. Reproduced from ref. 120 with permission from the American Chemical Society, copyright 2011.

The affinity of weak polyelectrolyte coated oxide particles to the oil–water interface can be controlled by the degree of dissociation and the thickness of the weak polyelectrolyte layer.^120^ To demonstrate this, weak polybase poly(allylamine hydrochloride) has been selected for the surface modification of oppositely charged alumina and silica colloids. Highly stable emulsions can be obtained when the degree of dissociation of the weak polyelectrolyte is below 80%. Cryo-SEM visualization showed that the regularity of the densely packed particles correlates with the degree of dissociation (Fig. 6).

Silica–poly(allylamine hydrochloride) particles arrange themselves in a monolayer which partially consists of some aggregates below pH 9.2. Above this pH value, flocculation of particles takes place; consequentially, the droplet shell consists almost entirely of particle aggregates.

Comparing to LbL deposition and colloidosome formation, polymerisation approaches for shell formation are more simple and robust, however they lack the possibility to make a PCM capsule shell possessing several functionalities like increased thermal conductivity, elasticity, targeted delivery and affinity to the surfaces of the heat transporting components (*e.g.*, metals) of the macroscale heat capacitors. Additionally, doping of the LbL shell with nanoparticles significantly increases its stiffness and resistance to deformation.^121^

However, micro and nanocapsule polymerisation using an emulsion template is at a more advanced stage of research than LbL for the fabrication of PCM-loaded capsules. It is also feasible for industrial scale-up.^122^ Traditional emulsion polymerisation is a type of free radical polymerisation, initiated by radicals which enter monomer droplets.^123^ By producing a miniemulsion, monomer droplets become much smaller (around 100 nm compared to 1–10 μm). This gives a far greater surface area, and therefore a better chance of radical initiation. The miniemulsion therefore provides an enormous number of parallel reactions taking place inside 10^18^–10^20^ nanodroplets.^124^ Other important types of polymerisation for encapsulation are interfacial polymerisation, *in situ* polymerisation, polycondensation and polyaddition.^48^ Methods for polymer shell formation, not directly related to polymerisation, are solvent evaporation and coacervation.^35^

Many polymer materials can be used to fabricate capsules employing simple polymerisation step.^125^ These include differing glass transition and melting temperatures, and they may be hydrophilic, hydrophobic or amphiphilic. Materials for encapsulation should be compatible with the PCM, have a higher *T*_M_ than the PCM and be stable to volume/pressure changes caused by melt–freeze cycles.

The selection of shell materials and structure of the composite PCM capsules is also very important for PCM stability and performance. As an example of lifetime requirements, PCMs incorporated into a building wall for air conditioning requires a life of at least 20 years. Assuming one melting/freezing cycle per day, the material must be stable for around 7300 cycles. An interesting potential solution to extend stability is the use of self-healing capsules. There are several examples in the literature of self-healing capsules which contain shell monomers in the core (*e.g.* diisocyanate in the core of a polyurethane capsules^126^). Additionally, self-healing effect can be achieved by combining polymerisation and LbL assembly methods for formation of PCM capsule shell. However, the combination of PCMs and self-healing materials has not yet been explored. The development of multi-compartmental capsules^127^ may also be of interest in order to use a combination of both organic and inorganic PCMs in one capsule for multitemperature heat storage, along with other beneficial materials such as corrosion inhibitors. Capsules containing multiple active materials are fabricated using multiple emulsions as a template. Zarzar *et al.* recently produced a simple one-pot method to fabricate complex three and four phase emulsions, demonstrating controllable and reconfigurable morphologies.^128^

## 3. Encapsulation of organic PCMs

As mentioned above, containment is crucial in giving PCMs the desirable properties for a wide-scale use. Micro- or nano-capsules can have different shapes such as spherical, tubular or an irregular one. An early example of an encapsulated PCM was a simulation by Theunissen and Buchlin,^129^ who determined a PCM storage system would require a volume 5 times less than that of a rock bed, in order to store an equal amount of energy. The encapsulation process for that study simply consisted of a large tank. Modern advances in emulsion and polymerisation chemistry allow for the fabrication of PCM capsules at the nanoscale, improving thermal characteristics.

### Polymer capsules loaded with organic PCMs

The most successful techniques for the fabrication of PCM-loaded capsules have been reactions of miniemulsion, *in situ* and interfacial polymerisations. During *in situ* and interfacial polymerisation, a polymer shell is formed at the oil/water interface, rather than within emulsion droplets. *In situ* polymerisation refers to systems in which the monomer is present only in one emulsion phase, whereas for interfacial polymerisations monomers are present in both phases. The morphology, physico-chemical and thermal properties of encapsulated organic PCMs are dependent on various synthetic parameters: stirring rate, content of emulsifier, content and type of initiator, core/shell weight ratio, shell/initiator weight ratio, polymerization temperature, polymerization time, *etc.* It is hard to predict the most appropriate combination of synthetic parameters for particular PCM-shell systems, requiring much experimentation to produce the most favourable properties. Typical monofunctional polymer shells for encapsulation of organic PCMs are polystyrene, polymethyl methacrylate (PMMA), urea–formaldehyde, melamine-formaldehyde and polyurethane.

**Fig. 7:**
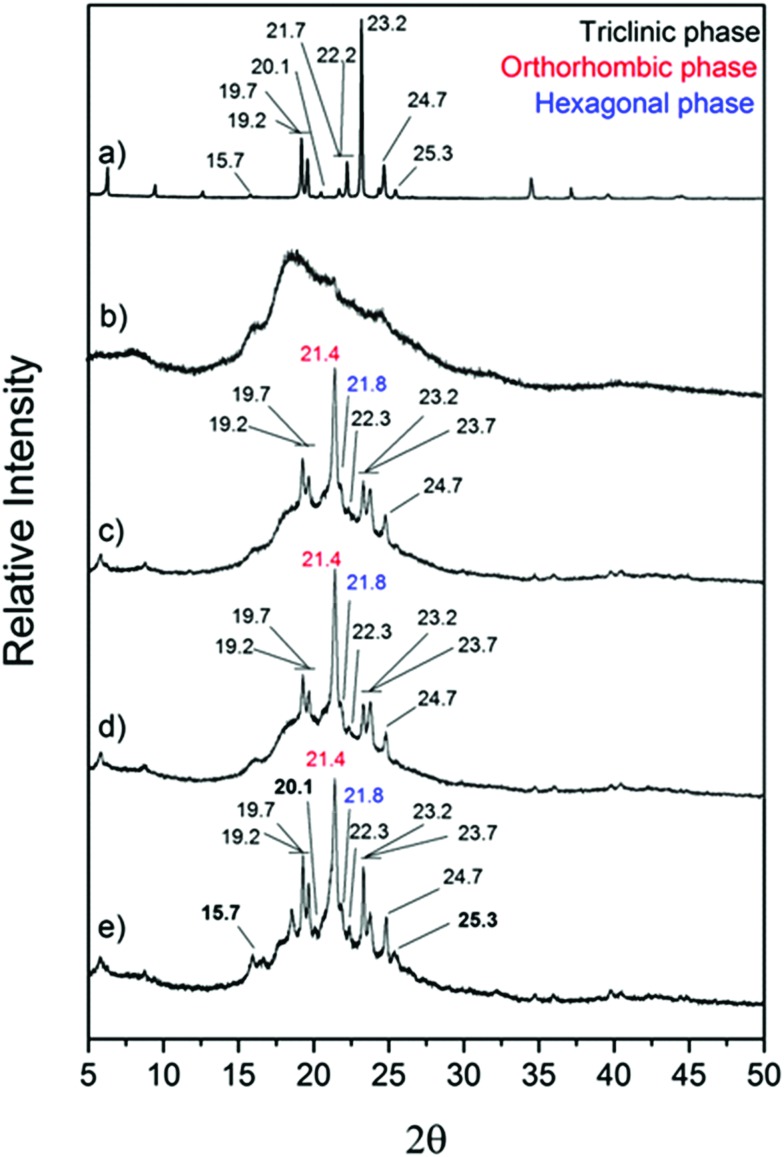
X-ray diffractograms of (a) bulk *n*-docosane, (b) polyurethane shell (hollow microcapsules), *n*-docosane loaded into (c) 10 m microcapsules, (d) 4 μm microcapsules and (e) 2 μm microcapsules. Reproduced from ref. 130 with permission from the John Wiley and Sons, copyright 2015.

Felix *et al.* have fabricated docosane loaded capsules using polyurethane as shell material, controlling size by varying homogenisation speed.^9^,^130^ The capsules were stable over at least 100 heat uptake/release cycles. An interesting effect was that the smaller capsules displayed higher latent heat compared with larger capsules. This shows the benefits of reduced capsule size, resulting in improved thermal performance. Boosting heat transfer to the core material contributes to increased latent heat. Another effect found with decreasing capsule size has been the distinct appearance of multiple crystalline phases during freezing (Fig. 7). Spatial confinement clearly has a significant effect on PCM behaviour.^130^

The diffractogram of polyurethane shell (Fig. 7b) shows two broad peaks, indicating low crystallinity of the shell itself. The encapsulated docosane demonstrates differing crystallinity when capsule size is altered. There are two amorphous peaks, previously seen in the polyurethane shell in the range of 15°–16° and between 16°–25°. The characteristic peaks, which are associated with the triclinic phase of docosane, appear at 19.29°, 19.70°, 22.31°, 23.28°, 23.79° and 24.79°. Face-centered orthorhombic phase appears as a sharp peak at 21.42°; the hexagonal packed phase appears at 21.80° as a small peak. Capsules of 2 μm size (Fig. 7e) display the previously mentioned peaks, which are noticeably more defined. Here, encapsulated docosane also has additional peaks associated with the triclinic phase at 20.07°, 25.37° and 15.95° which are observed in the bulk docosane, but not within the larger capsules of 10 μm (Fig. 7c) and 4 μm (Fig. 7d). This is in agreement with the higher melting and crystallization enthalpies (Δ*H*_m_ = 79 J g^–1^ and Δ*H*_c_ = 88 J g^–1^) found for the smaller capsules which is related to higher crystallinity. Capsules with 10 μm and 4 μm do not have the additional peaks from the triclinic phase and showed smaller melting and crystallization enthalpies (Δ*H*_m_ = 47 J g^–1^ and Δ*H*_c_ = 48 J g^–1^ for capsules with 10 μm and Δ*H*_m_ = 53 J g^–1^ and Δ*H*_c_ = 53 J g^–1^ for capsules with 4 μm).^130^

Two reports have noted monomer effects on encapsulation using toluene diisocyanate along with amines as crosslinkers for polyurea shell formation.^131^,^132^ Amines used have been ethylene diamine, diethylene triamine and Jeffamine (amine-terminated polyoxypropylene). Longer chain amines formed capsules of larger diameter, along with better coverage of the core material. The authors state this is due to the hydrophilic amines requiring migration into the oil phase to react with the diisocyanate. Monomer selection is therefore an important factor to consider when fabricating PCM loaded capsules. Alkan *et al.* used emulsion polymerization to encapsulate eicosane in polymethyl methacrylate.^133^ It has been also found that type of shell and core materials affect the capsule diameter, encapsulation ratio and heat storage capacity.

A polymer shell can also be used for encapsulation of paraffin mixtures and eutectics.^134^ Four alkane mixtures (heptadecane–tetracosane, octadecane–nonadecane, nonadecane–tetracosane and icosane–tetracosane) have been encapsulated into polymethyl methacrylate shell *via* emulsion polymerisation. DSC thermograms of encapsulated paraffin eutectics had transition temperatures similar to that of the bulk eutectics. The eutectic composition of the mixture is therefore preserved during the encapsulation process.

LbL assembly technique has been applied to build up ultrathin nanostructured shells for PCM capsules.^135^ The oppositely charged strong polyelectrolytes poly(diallyldimethylammonium chloride) and poly(4-styrenesulfonic acid) sodium salt have been employed to fabricate three multilayer shells with a thickness of ∼10 nm on emulsified octadecane droplets. Using bovine serum albumin as the surfactant, polyelectrolyte encapsulated octadecane spheres with a size of ∼500 nm were obtained with good shell integrity, high octadecane content (91.3 wt%), and thermal stability of 5 cycles.

Nanocapsules with polymer shell are structurally more stable than microcapsules as shown by Sukhorukov *et al.*^114^ Many authors have employed miniemulsion method for fabrication of organic PCM nanocapsules. Zhang *et at.*, Fan *et al.*, Konuklu *et al.* have employed miniemulsion polymerisation for docosane encapsulation into 100–150 nm melamine formaldehyde nanocapsules.^136^–^138^ The same method but with PMMA shell has been used by Chen *et al.* and Sari *et al.* for paraffins encapsulation into 150 nm capsules.^139^,^140^ Yang *et al.* synthesised poly(methyl methacrylate), poly(ethyl methacrylate) and polystyrene capsules containing tetradecane using *in situ* polymerisation, and found the acrylate capsules performed far better with regards to heat storage.^141^ Zhang *et al.* have also made poly(methyl methacrylate) and poly(ethyl methacrylate)capsules, but used sonication in order to perform a miniemulsion polymerisation.^95^ This resulted in nanocapsules of 100–150 nm in size and encapsulated octadecane with a high efficiency of 89–95 wt%. Both researchers noted that supercooling of the paraffin was reduced once encapsulated. Ultrasound has been applied by Fang *et al.* to reduce the size of *n*-dotriacontane/polystyrene capsules below 200 nm.^142^ The duration of the ultrasonic treatment does not have linear influence on the capsule size. First, the capsule size is reduced below 160 nm but after continued sonication, average size increases to 200 nm. This effect is caused by aggregation of PCM droplets due to the overall heating of the reaction mixture at prolonged sonication time.

**Fig. 8:**
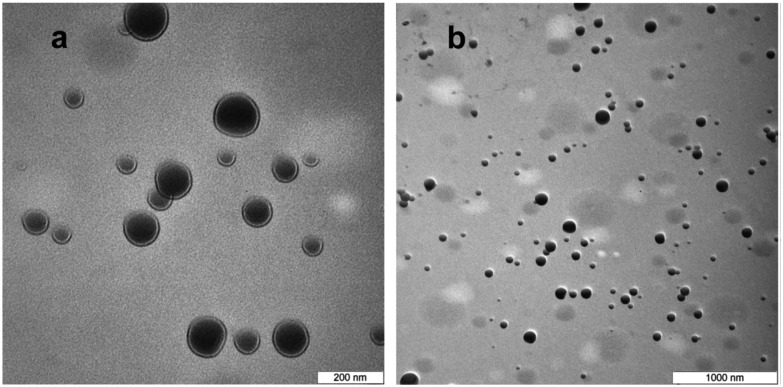
TEM images of the *n*-octadecane/polystyrene/(PMMA) capsules at (a) 100k magnification and (b) 30k magnification. Reproduced from ref. 143 with permission from the Elsevier, copyright 2014.

Composite polystyrene/PMMA shell was successfully employed for synthesis of highly stable PCM nanocapsules with octadecane as PCM core.^143^Fig. 8 shows TEM images of the *n*-octadecane/polystyrene/PMMA nanocapsules. It can be seen that most of the nanocapsules have regular spherical shape. The shell/core morphology is clearly observed. Dark sections of the images show *n*-octadecane was located inside the shell, proving successful encapsulation. The nanocapsules are stable after 360 heating/cooling cycles and have phase change enthalpies of 107.9 J g^–1^ (melting) at 29.5 °C and 104.9 J g^–1^ (crystallization) at 24.6 °C.

Other methods such as interfacial polymerization and sol–gel precipitation have been used to a lesser extent.^144^,^145^ Wang *et al.* nanoencapsulated an eicosanoic–stearic acid eutectic in a PMMA shell by UV initiated emulsion polymerisation, finding particle size and the size distribution both decreased with agitation speed, use of cross-linking agent and reduction of monomer and initiator concentration. The nanocapsules of ∼46 nm diameter had good thermal stability and displayed decreased supercooling compared to the bulk eutectic.^146^

### Organic PCM-loaded capsules with inorganic shell

The main drawback of organic shells used for encapsulation of organic PCMs is their very low thermal conductivity which, in addition to the low thermal conductivity of organic PCMs, results in very slow heat exchange with environment during heat uptake/release cycles, supercooling and overheating. To improve thermal conductivity, several attempts have been made to encapsulate organic PCMs into inorganic shells, mostly made of inert silica, alumina or clay materials.

Yin *et al.* have made a hybrid SiO_2_/polystyrene/poly(divinyl benzene) shell using a Pickering emulsion template, resulting in capsules of approximately 100 μm diameter.^125^ Modified SiO_2_ nanoparticles were used as stabiliser and, due to the presence of –C=C groups on their surface, become embedded in the shell by covalent bonding. Other researchers have reported the formation of a full SiO_2_ shell by hydrolysing tetraethyl orthosilicate (TEOS) to create an encapsulation precursor. This gave capsules of 8–15 μm and a high encapsulation efficiency up to 87.5 wt%.^147^ Silica nanocapsules containing octadecane and *n*-dodecanol core were synthesized by using TEOS as an inorganic source through a sol–gel process.^148^ The authors noted the capsule thermal conductivity was 0.621 W m^–1^ K^–1^ compared with 0.151 W m^–1^ K^–1^ for bulk octadecane. They also showed the silica shell has a conductivity of 1.296 W m^–1^ K^–1^, compared with polymers which are around 0.20 W m^–1^ K^–1^.^149^ Fu *et al.* used polystyrene–silica shell for nanoencapsulation of *n*-tetradecane.^150^ The resulting capsules have 150 nm size and 83.38 J g^–1^ latent heat. Addition of silica to the capsule shell by just 1 wt% increased its thermal conductivity by 8.4%. Similar composite nanocapsules can be obtained substituting polystyrene with polymethylmetacrylate.^151^

**Fig. 9:**
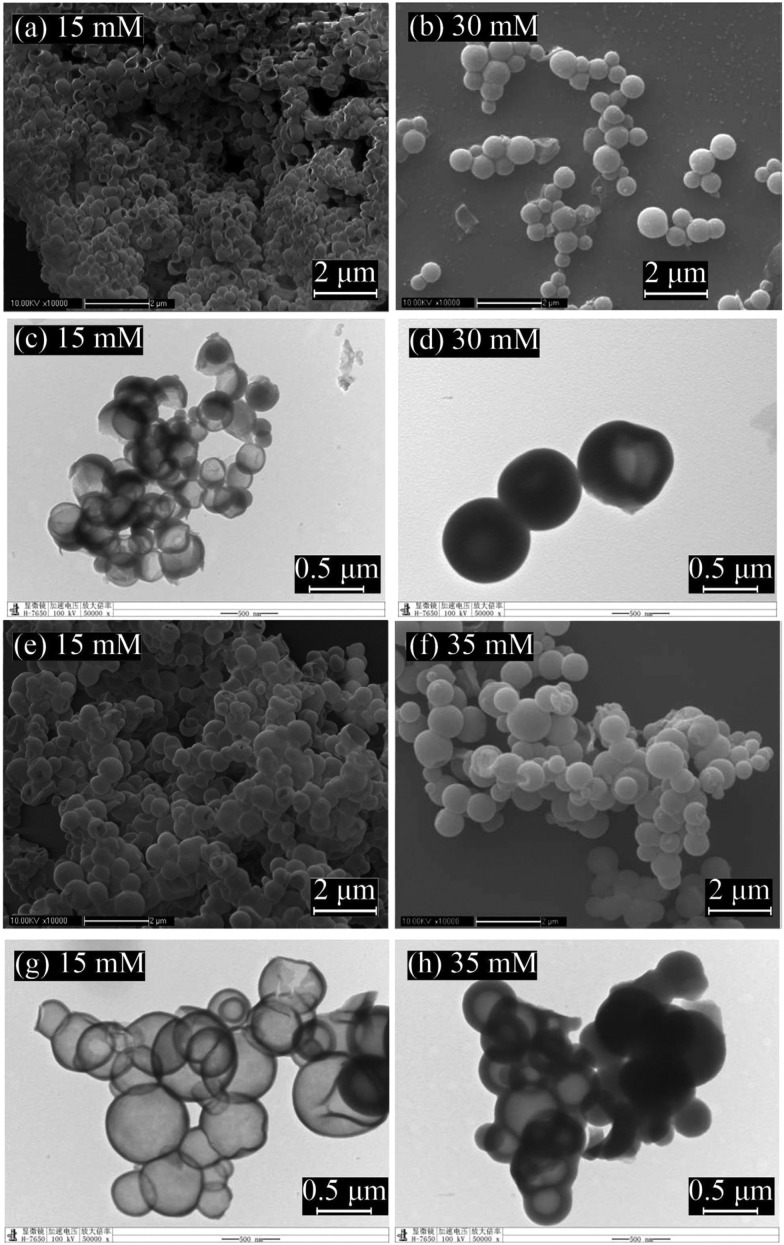
(a, b, e, f) SEM and (c, d, g, h) TEM images of the silica nanocapsules loaded with *n*-octadecane prepared at different CTAB concentrations. Reproduced from ref. 152 with permission from the Elsevier, copyright 2016.

Changing silica content and hydrolysis rate, the morphologies of the PCM-loaded capsules can be regulated from thin-shelled nanocapsules with bowl-like, hemispherical or spherical geometries to thin-shelled spherical nanocapsules and even mesoporous particles (Fig. 9).^152^ At low CTAB concentration (15 mM), bowl like or spherical nanocapsules with well defined core–shell structure are obtained, and the shell thickness is relatively low. However, at higher CTAB concentration (30 or 35 mM), spherical nanocapsules with thicker shells are acquired. The PCM content in mesoporous silica nanocapsules is 30 wt% less than in hollow ones. Due to the confinement of *n*-octadecane in nanosized organosilica shells, the homogeneous nucleation is suppressed, resulting in a notable shift of crystallizing points. Encapsulated *n*-octadecane crystallises *via* shell induced heterogeneous nucleation and thicker organosilica shell induces the heterogeneous nucleation better. After 500 melting/solidifying cycles, the nanocapsules maintained their phase transition properties perfectly, indicating high thermal reliability.^153^ In addition, the hydrophobicity of the organosilica shell materials can be tuned by changing the volume ratios of silane precursors. The theoretical modelling showed that the mobility of the encapsulated *n*-octadecane is restrained within constrained SiO_2_ shell.^154^

Besides improved thermal conductivity, inorganic shell materials can also provide flame-retardant properties to the encapsulated organic PCM. Demirbag *et al.* have reported a study on the thermal stability and flame-retardant properties of PCMs encapsulated into clay nanoparticles doped gelatine/sodium alginate shell.^155^ The capsules have been fabricated by the technique of complex coacervation using gelatine and sodium alginate as the shell and *n*-eicosane as PCM core. Nanocomposite structure of the capsule shell demonstrates improved flame retardant properties of cotton fabrics when treated with PCM-loaded capsules. Similar flame-retardant effect has been achieved for gelatine/gum arabic and gelatine/sodium alginate shell doped with alumina nanoparticles.^156^

### Nanostructured shells from C-based materials

Although inorganic shells demonstrate thermal conductivity enhancement, their long-term stability is brought into question due to their brittleness. Despite good thermal and chemical stability, they may fracture due to stress formed by PCM volume change during melting. Hence, the next generation of shell materials for encapsulation of organic PCMs should combine both high thermal conductivity inherent to the inorganic shell and elasticity of the polymer shell. This can be done using nanostructured carbon-based materials as main shell component.

An excellent example of a multifunctional shell for paraffin encapsulation was demonstrated by Zheng *et al.*^157^,^158^ The capsules are comprised of encapsulated paraffins and graphene oxide/carbon nanotube hybrids (GO–CNT) as core and shell material, respectively. Multiform carbon nanotubes stabilize GO capsule shell to resist volume-change induced rupture during heat uptake/release and enhance the thermal conductance of encapsulated paraffins.

**Fig. 10:**
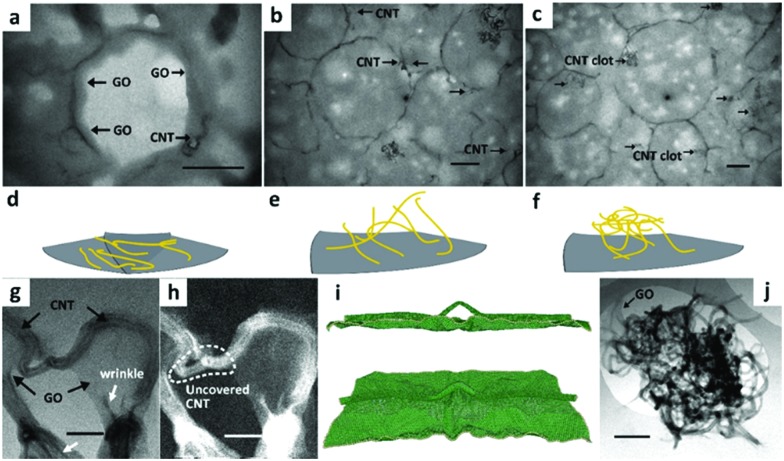
Structural and morphological characterization of docosane/GO–CNT microcapsules. TEM images of ultrathin sections from ultramicrotomy showing (a) the CNTs are completely adhered on GO layers, (b) partially adhered and partially inwards extended, and (c) even led to clots embedded in docosane, respectively. (d), (e) and (f) Show schematic models of the three configurations of CNTs within docosane/GO–CNT microcapsules. The grey lamellar and yellow wires represent the GO sheet and CNTs, respectively. TEM images of GO–CNT hybrids in bright field imaging mode (g) and high-angle annular dark-field imaging mode (h) are shown. Molecular dynamics simulation of the GO–CNT assembly show side view (i top) and tilted top view (i bottom) of a snapshot during the relaxation process. The wire-like nanotube and silk-like GO can be distinguished. Besides, a CNT clot (j) is adhered by GO sheets. Scale bar: (a–c) 500 nm; (g and h) 50 nm; (i) 5 nm; (j) 200 nm. Reproduced from ref. 157 with permission from the American Chemistry Society, copyright 2016.

The GO–CNT hybrids along with CNT clots (Fig. 10) were formed under pulsed tip-sonication and then directly applied in the following ultrasound-induced emulsification with melted docosane. Detailed observations at the sections of ultramicrotomed docosane/GO–CNT have unclosed the multiform CNTs in capsules: completely horizontal adherence on GO layers (Fig. 10a and d), partial adherence and partial inwards extension (Fig. 10b and e), and clot attached to the inner wall of capsules (Fig. 10c and f). The partial coverage is common among the CNTs longer than the persistence length, they are modeled as semi-flexible chain with configuration of partially adhering on GO layer and partially penetrating into the interior (Fig. 10e).

The original shape of docosane/GO–CNT solid powder was retained at temperatures higher above the melting point of docosane; whereas the unprotected docosane deformed into liquid quickly. There is little change in docosane/GO–CNT thermal properties after 100 thermal cycles without notable supercooling. The average latent heat of encapsulated docosane remained around 240.8 J g^–1^, leading to an encapsulation ratio of paraffin as high as 96.7 wt% by comparing with the enthalpy of bulk state.

Through changing the cooling rate of original emulsions, it was possible to control the inner structure of the organic PCM capsules.^158^ The special geometry of PCMs is often hidden beneath the capsule shell and it influences whole system performance on molecular level. Hollow and solid structures of the PCM core determine the thermal properties of energy capsules with nanocarbon shell. The pronounced C–H···π interaction of hollow PCM core might be responsible for more stable encapsulation and greater heat diffusivity of melted PCMs, as compared with solid PCM core with weak PCM-shell interaction. Graphene nanosheets can be used to increase thermal conductivity in nanoencapsulated PCMs, however this leads to an undesirable rise in viscosity.^159^ The dramatic viscosity growth, up to over 100-fold at the highest loading, deteriorates significantly the intensity of natural convection, which has been identified as the dominant mode of heat transfer during melting of PCM core. The loss in natural convection was found to overweigh the enhancement in heat conduction, thus resulting in decelerated melting.

**Fig. 11:**
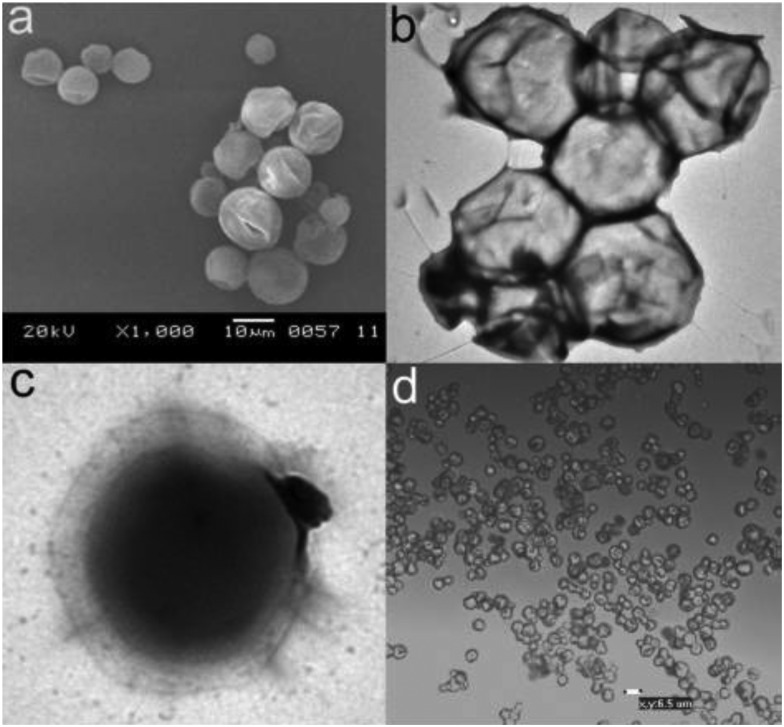
(a) SEM, (b and c) TEM, and (d) optical microscopic images of the eicosane/EC/MC capsules prepared with a 9 wt% polymer content. Reproduced from ref. 160 with permission from the American Chemical Society, copyright 2011.

Eicosane-loaded capsules with a 2 : 1 ratio of ethyl cellulose (EC) : methyl cellulose (MC) as shell material have been prepared through a novel liquid to solid encapsulation process.^160^ The obtained water suspension of microspheres was cooled below the melting point of eicosane, thus solidifying the core material. Solidification is accompanied by a decrease in volume, thus accounting for the observed dents in the capsules (Fig. 11).

The indentation of the wall of the capsules correlates well with the maximum interaction between eicosane molecules and the ethoxy moieties of the EC polymer. DSC measurements have demonstrated the increase of the absolute enthalpy value during the crystallization of the eicosane-loaded EC/MC capsules as compared to the pure eicosane, implying that the encapsulated eicosane molecules release more energy upon solidification. With the presence of 9 wt% of the EC/MC polymer, the capsules demonstrated a 24% increase in heat absorption and a 29% increase in heat release. It is likely that the interaction between the EC/MC shell and the encapsulated eicosane causes a different phase transition path for the encapsulated eicosane than for free eicosane. The new path contains multiple broad overlapped transition steps, releases more energy than the two-step crystallization of the pure eicosane and gives crystals with a different XRD pattern. An encapsulation process that allows for a good specific interaction between the polymer shell and the PCM is therefore crucial.

There are several other examples of using nanocellulose-based materials as PCM shells in form of Pickering emulsions. Capron *et al.* have produced Pickering emulsions of hexadecane in water stabilized with bacterial cellulose nanocrystals.^161^ High stability has been observed without any droplet size change after centrifugation. Svagan *et al.* have reported chemically cross-linked nanocellulose-based capsules with a hexadecane core.^162^ Although no thermal properties were recorded, the capsules had excellent elastic properties which are ideal to withstand volume changes. Li *et al.* have encapsulated RT25HC paraffin (Rubitherm Technologies GmbH) in nanocellulose, employing a Pickering emulsion method combined with ultrasonication.^163^ Nanocellulose diffuses into the paraffin core and stabilizes water/paraffin interface, forming a shell around paraffin droplets. Excess of nanocellulose forms a three-dimensional network with embedded energy capsules thus preventing their coalescence. The PCM composite has showed a solid content of paraffin of 72 wt%.

## 4. Encapsulation of inorganic PCMs

**Fig. 12:**
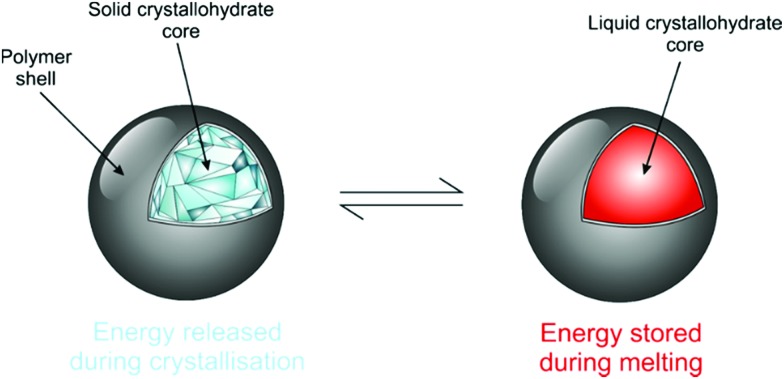
Cartoon showing ideal capsule behaviour for the salt hydrate core. Reproduced from ref. 173 with permission from the Royal Society of Chemistry, copyright 2017.

Salt hydrates are difficult to encapsulate due to their hydrophilicity, tendency to alter water content and surface polarity.^164^,^165^ Further added to these issues is their general chemical instability, which is well documented.^10^ Encapsulation can promote improved stability due to effects such as confinement of stoichiometry and improved heat transfer to decrease supercooling. It also acts as a barrier to prevent water loss from the crystallohydrate core, maintaining its thermal characteristics (Fig. 12). The enhanced energy storage properties of inorganic PCMs compared with organic ones make further research into their encapsulation highly beneficial.

Several chemical encapsulation methods have been reported in a review by Milian *et al.*^166^ Inverse Pickering emulsion, interfacial polymerisation and solvent evaporation–precipitation are the most common chemical methods described for the encapsulation of inorganic PCMs although there are relatively few examples to date.

In 2001, Gröhn *et al.* encapsulated chloroauric acid trihydrate within a dendrimer system, although this was not for energy storage purposes.^103^ To the best of the authors’ knowledge, the first attempted core–shell capsules containing salt hydrates specifically for energy storage were developed by Sarier and co-authors.^167^ They used a mix of PEG1000, hexadecane and sodium carbonate decahydrate (Na_2_CO_3_·10H_2_O) as core material encapsulated in an urea–formaldehyde shell. They noted the phase change behaviour resembled that of hexadecane and Na_2_CO_3_·10H_2_O did not contribute. The same authors also developed a method of trapping PCM micelles inside a polyurethane foam.^168^ One of the PCM combinations they used was octadecane and Na_2_CO_3_·10H_2_O. However, they found Na_2_CO_3_·10H_2_O acted merely as a blowing agent due to its water content, and did not contribute to the latent heat. As these researchers have been using O/W emulsions rather than W/O, the resulting capsules contained very little salt hydrate.

Salaün *et al.* have encapsulated Na_2_PO_4_·12H_2_O in a polyurea/polyurethane shell.^169^ They employed the solvent evaporation technique, dissolving cellulose acetate butyrate in a volatile solvent (chloroform). As the volatile solvent evaporates, polymerisation of cellulose acetate butyrate occurs. They used methylene bis(phenyl 1 isocyanate) as the crosslinker to form the polyurea shell. They have not demonstrated thermal energy storage characteristics or accurate size measurements (only stating capsules were >1 μm), although they did show the capsules contained a large amount of salt, up to 79 wt% of loading. In further research, they investigated the influence of solvent on capsule properties. They found that changing the solvents for the dispersed and continuous phases had a profound influence on the characteristics of capsules. Using chloroform as dispersed phase solvent instead of acetone facilitated the full coverage of the core with shell material. Using toluene as continuous phase instead of carbon tetrachloride reduced coacervation rate and produced better defined capsules. DSC results revealed that most of the formed capsules had incomplete crystallisation processes, meaning that by the 2nd thermal cycle, no latent heat storage was possible. The most successful capsules used toluene and chloroform as solvents with a latent heat of melting of 140.4 J g^–1^. The latent heat of crystallisation was only 48.9 J g^–1^, suggesting that long-term stability is not possible.

Similar work has been done by Liu *et al.*, on the encapsulation of sodium thiosulfate pentahydrate into microcapsules with silica shell.^170^ The authors demonstrated successful encapsulation of inorganic PCM into silica shell by sol–gel method with high encapsulation rate (94.65 wt%). However, the stability of the capsules during the heat uptake/release cycles was not demonstrated.

Graham *et al.* have demonstrated a simple method to nanoencapsulate magnesium nitrate hexahydrate, employing an *in situ* miniemulsion polymerisation with ethyl-2-cyanoacrylate as monomer.^171^ Using sonication to prepare miniemulsions improved the synthesis by reducing the amount of surfactant required as stabiliser.

**Fig. 13:**
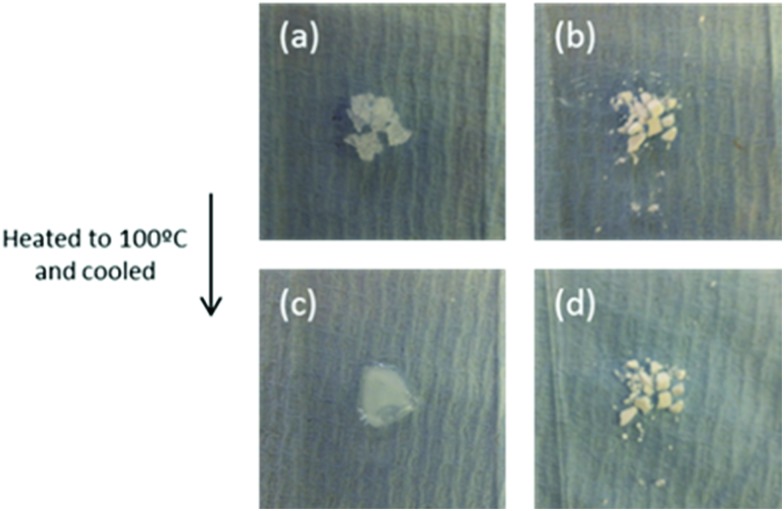
Bulk Mg(NO_3_)_2_·6H_2_O (a and c) and nanoencapsulated salt hydrate (b and d) before heating to 100 °C (top), and after letting them cool back to room temperature (bottom). Reproduced from ref. 171 with permission from the Royal Society of Chemistry, copyright 2016.

Before melting, pure Mg(NO_3_)_2_·6H_2_O (*T*_M_ = 89 °C) is a crystalline solid (Fig. 13). After melting, it recrystallizes to the solid shown in Fig. 13c. This solid is surrounded by water, showing a volume change occurs during phase transition. The recrystallized solid forms a compact block, which prevents free diffusion of water vapour. In contrast to the bulk material, nanoencapsulated salt hydrate (Fig. 13b and d) shows no volume increase or change in appearance before and after heating to 100 °C. This indicates its chemical and structural stability at the transition temperature. The absence of leakage shows the salt hydrate is fully protected by encapsulation from the outside environment, helping prevent changes in salt hydrate composition in the nanocapsule core. These observations are consistent with their DSC results, which showed the Mg(NO_3_)_2_·6H_2_O was stable over at least 100 cycles, remaining fully hydrated due to the addition of extra water to the salt hydrate core, a principle previously demonstrated in macroscale systems.^172^

**Fig. 14:**
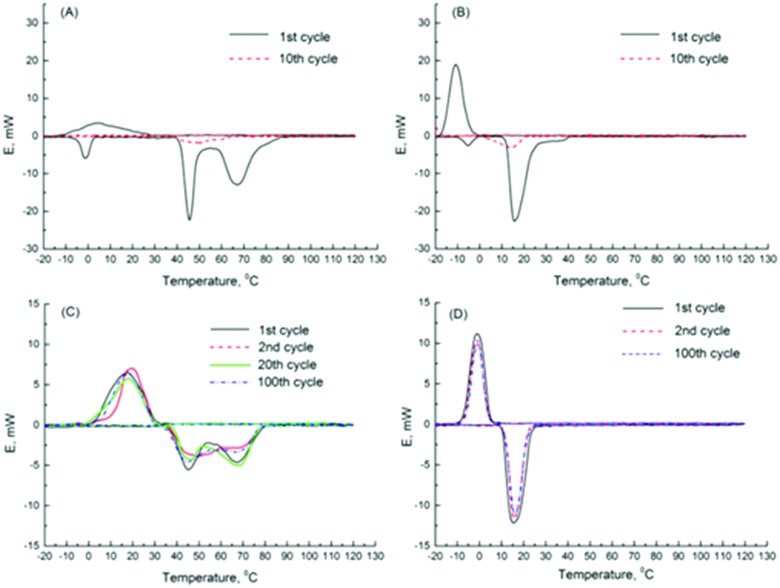
DSC data for (A) 1 : 1 wt% Mg(NO_3_)_2_·6H_2_O : Na_2_SO_4_·10H_2_O bulk mixture, (B) 1 : 2 wt% Mg(NO_3_)_2_·6H_2_O : Na_2_SO_4_·10H_2_O bulk mixture, (C) encapsulated 1 : 1 wt% Mg(NO_3_)_2_·6H_2_O : Na_2_SO_4_·10H_2_O and (D) encapsulated 1 : 2 wt% Mg(NO_3_)_2_·6H_2_O : Na_2_SO_4_·10H_2_O. Reproduced from ref. 173 with permission from the Royal Society of Chemistry, copyright 2017.

Their follow-up paper describes encapsulation of two crystallohydrates (Mg(NO_3_)_2_·6H_2_O and Na_2_SO_4_·10H_2_O) and their eutectic mixture.^173^ DSC results demonstrated high thermal stability of nanoencapsulated single and mixed crystallohydrates, which remained unchanged after 100 thermal cycles (Fig. 14). Encapsulation of the crystallohydrate mixtures prevents the loss of water during prolonged thermal cycling, resisting changes to the *T*_M_ and latent heat, and also reduces supercooling (Fig. 14C and D). After encapsulation, the 1 : 2 Mg(NO_3_)_2_·6H_2_O : Na_2_SO_4_·10H_2_O eutectic ratio (Fig. 14D) was maintained, with one well defined phase transition peak at *T*_M_ = 15.4 °C and *T*_F_ = –1.1 °C and reduced supercooling of Δ*T* = 16.5 °C. The transition is stable over >100 heat uptake/release cycles and has latent heat capacity of 126.8 J g^–1^, which results in 67 wt% encapsulation efficiency. The effect of nanoencapsulation on salt hydrate mixtures is similar to its effect on single crystallohydrates, producing thermally stable energy capsules. The authors demonstrated that additive mixtures of nanocapsules containing single crystallohydrates have good potential for the design of multi-temperature heat storage systems containing energy capsules with different PCM cores sensitive to multiple transition temperatures.

Huang and collaborators fabricated capsules with Na_2_PO_4_·12H_2_O as core material and methyl methacrylate as monomer along with ethyl acrylate as crosslinker.^164^ The shell has been made by a suspension polymerisation combined with solvent evaporation. They found that, upon encapsulation, the PCM was partially dehydrated to form Na_2_PO_4_·7H_2_O which resulted in an increase in melting temperature from 36 °C to 51 °C. They also made a follow up article, where they compared their PMMA capsules to urea formaldehyde ones.^174^ The PMMA capsules demonstrated much better thermal characteristics, while urea formaldehyde capsules have very broad melting temperature peak at 41 °C, which is unsuitable for practical applications when a narrow temperature range is required. Importantly, upon encapsulation the thermal conductivity increased from 1.01 W m^–1^ K^–1^ for pure Na_2_PO_4_·12H_2_O to 1.426 W m^–1^ K^–1^ for the encapsulated Na_2_PO_4_·7H_2_O.^166^

Platte *et al.* have encapsulated different mixtures of sodium sulphate, sodium phosphate and sodium carbonate which were hydrated by dissolving in water.^165^ They used a surface-thiol Michael addition polymerisation using ORMOCER polymers as shell material, which are biodegradable inorganic–organic hybrid polymers developed by Fraunhofer ISC, Germany. ORMOCERs are impermeable to water, which is of great benefit to encapsulated salt hydrates to maintain the desired hydration state. Formed capsules are around 40 μm in diameter. Supercooling was still a problem, showing the capsules did not sufficiently improve heat transfer. Schoth *et al.* have developed a surfactant free method to encapsulate sodium sulphate decahydrate.^175^ They utilised the Pickering emulsion technique to create the initial emulsion, resulting in polyurethane nanocapsules with an average size of 750–1000 nm. It was also shown that Na_2_SO_4_·10H_2_O could be encapsulated up to 20 wt% (its solubility limit in water). Hassabo *et al.* prepared PCM capsules with a poly(ethoxysiloxane) shell. The capsules contained crystallohydrates and a Pickering emulsion template of silica nanoparticles was used.^176^ Silica dispersions mixed with inorganic salts in various ratios were dissolved in toluene, and pH was adjusted to pH = 1 using HCl. Encapsulation had minimal effect on the *T*_M_, however latent heat was low.

An interesting approach for salt hydrate encapsulation is the use of nanobowls, consisting of SiO_2_ matrix impregnated with Na_2_SO_4_·10H_2_O.^177^ The formation of the unusual bowl shape is due to non-synchronous rotation of droplets caused by viscosity differences between the liquid and solid phases. The SiO_2_ matrix improved heat conductivity of the PCM, as well as reducing phase separation. The sample also had high latent heat of 180.7 J g^–1^ which was relatively unchanged after 60 cycles. However, supercooling was only marginally reduced.

Strong interface interactions between core and shell materials can influence on the heat uptake/release of the capsules, similar to that observed for encapsulation of organic PCMs.^159^ Another factor is the nature of shell material, which presents different heat conduction and natural convection contributing to heat transfer as much as melting and glass-temperature.^178^

## 5. Cutting edge systems for thermal energy storage based on encapsulation

In this section of our review, we concentrate on three fields that have potential to be greatly accelerated by incorporating nanoencapsulated PCMs including heat-transfer fluid and PCM slurry, energy harvesting and conversion, and 3D printed functional composite. A few examples of microcapsule use for thermo-regulated textiles^179^,^180^ and building envelope materials^181^ have been reported but they are beyond the topic of this review. The toxicity issue of nanomaterials to the environment^182^ and human health is not yet fully understood,^183^ so these effects must be studied with regards to PCM nanocapsules before their widespread use.

### Heat-transfer fluid and PCM slurry

A heat-transfer fluid (HTF) is often utilized to transfer thermal energy from a hot source to a cold one, to maintain the stability of device performance and industrial safety.^184^ Of importance to determine the heat-transfer efficiency is the heat capacity of the fluid.^184^,^185^ PCMs exhibit a high latent thermal energy capacity near the melting point. Thus, properly selected PCMs can be dispersed in the liquids and enhance their heat capacity at the specific temperature range,^186^ leading to an enhanced heat-transfer capability of the fluid.^187^,^188^ The combination of HTF and dispersed PCMs is called a PCM slurry. Han *et al.*^189^ theoretically demonstrated the impact of volume fraction (*Φ*) and latent heat of dispersed PCMs to the effective specific heat (*C*_eff_) of the HTF, as outlined in the equation:*C*_eff_ = *C*_0_ + *ΦH*_PCM_/Δ*T*where *C*_0_ is specific heat of the base nanofluid, *H*_PCM_ is the latent heat of the dispersed PCM per unit volume, and Δ*T* is the temperature difference between the *T*_M_ and *T*_F_ of the PCM. The performance of microencapsulated PCM is not reliable after repeated cycling, because the fluid's viscosity is high in the case of high volume fractions and crushing between large particles is unavoidable during pumping. The development of nanoencapsulated PCM slurries is important because (i) they are structurally more stable than the micron counterpart,^114^,^190^,^191^ (ii) the thermal fluids containing nanoencapsulated PCMs show low flow drag, and thus low viscosity and (iii) the non-conformity of the close contact between nanoparticles and the HTF reduces the resistance of heat transfer.^192^

**Fig. 15:**
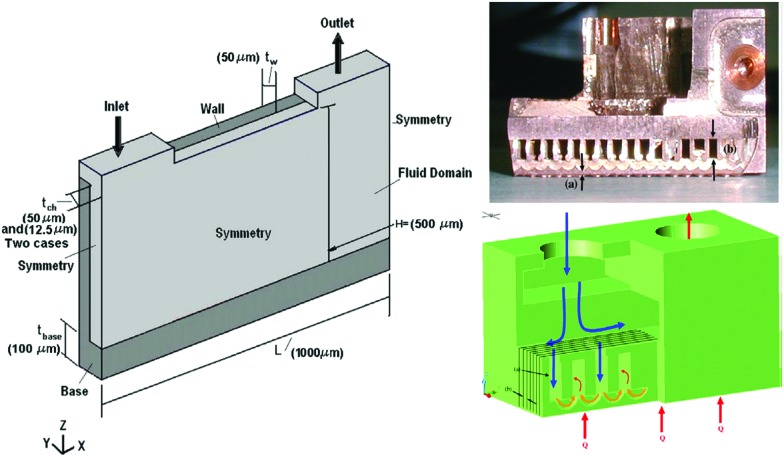
Design concept and cross-sectional view of microchannel heat exchanger. Reproduced from ref. 192 with permission from the Elsevier, copyright 2013.

Advanced microchannel heat sinks (Fig. 15) have become popular in recent years due to overheating generated by the miniaturization and high integration of electronic components or systems.^193^ As a novel working medium, the nanoencapsulated PCM slurry enhances both the thermal capacity and conductivity during phase transition process.^194^ A typical example is silica nanoencapsulated indium PCMs applied into poly-a-olefin (PAO) thermal fluid for high temperature (150–180 °C) microchannel heat exchange.^192^ The silica shells not only minimize the leakage of liquid indium but also prevent agglomeration of nanocapsules at high temperature. Experiments with the microchannel heat exchanger indicated that the optimized heat transfer coefficient of nanoencapsulated PCM slurry could reach 47 000 W m^–2^ K at a flow rate of 3.5 ml s^–1^. The magnitude of heat transfer coefficient represents a twofold improvement over that of single phase PAO. Seyf *et al.*^195^ have systematically investigated the influence of mass concentration and melting range of nanoencapsulated PCM, as well as Reynolds number on the thermal-hydraulic performance of a microchannel heat sink. They found that a combination of nanoencapsulated PCM and PAO fluid can enhance the thermal performance of the device in the aspects of lowering entropy generation and decreasing temperature uniformity. However, extra attention should be paid to eliminate the pressure drop as a consequence of increased mass concentration. Like many other applications involving PCMs, high thermal energy storage capacity and heat transfer efficiency of the nanoencapsulated PCM slurry are only possible near the phase transition range (PTR). Wang *et al.*^186^ found that the heat capacity of a nanoencapsulated PCM slurry below the melting point would likely decrease with increased PCM mass fraction due to the lower sensible heat capacity of PCM than the base fluid, which limits the use of single PCMs for this application. A solution to this problem may be to employ multiple PCMs with different *T*_M_s in a cascaded approach, arranged in order of decreasing *T*_M_ with regards to the direction of HTF flow during charging. This has been shown to increase thermal efficiency, due to maintenance of a constant temperature difference between the PCMs and HTF.^196^ Qu *et al.* recently confirmed the enhancing role of nanoencapsulated PCM by computing the thermo-physical properties of slurries.^197^ In addition to the mass concentrations and Reynolds number, they also found higher heat transfer rates could be obtained by reducing the thermal boundary layer thickness at stagnation zone of slot jet impingement mode.

### Energy harvesting and conversion

Solar-thermal and electro-thermal conversion, where either solar irradiation or electrical energy is harvested and converted to heat for beneficial usage, has been widely used in daily applications. The primary evaluation criterion is the output temperature at a certain input power, encouraging research to increase the energy conversion efficiency of the device further. Due to their large LHS capacity over the PTR, ideal PCMs will facilitate a decrease in size of cooling systems. This concept is emerging for using micro/nanoencapsulated PCMs as additives/dopants to thermal conversion systems.^159^ However, the main challenge is to increase the conversion efficiency not only at the PTR but also at temperatures lower or higher than the PTR. The PCMs store a higher accumulative energy (latent heat + sensible heat) above PTR than that within PTR, while exhibiting a much lower specific heat capacity. In other words, the liquid PCMs above the PTR can release the heat back to the heat-generating systems in the absence of phase transition. Especially at low volume/mass fraction, the synchronous temperature increase of incorporated PCMs and heat-generating structures is widespread. The heat generated by the incorporated PCMs, therefore, offsets the convective heat dissipation occurring at the outer surface,^198^ especially for sponge structures with a porous network and large surface area constantly enabling rapid exchange of heat with the environment but meanwhile suffer from severe convective heat dissipation.

**Fig. 16:**
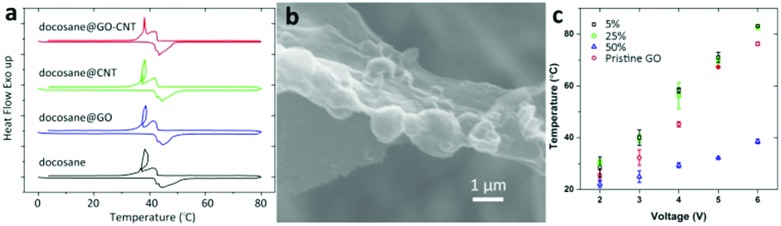
(a) DSC curves of bulk docosane and docosane encapsulated by GO, CNT and GO–CNT. (b) High-resolution SEM shows the capsules are embedded at basal plane of GO sheets, resulting in a “built-in” structure. (c) As a function of voltage, the balanced surface temperatures are demonstrated for composites containing 0, 5, 25 and 50 wt% docosane/GO–CNT capsules. Reproduced from ref. 158 with permission from the American Chemical Society, copyright 2017.

Based on the above considerations, Zheng *et al.* fabricated GO–CNT hybridized capsules containing long-chain alkane PCMs.^158^ The highly thermal conductive shell facilitates quick heat exchange between the confined alkanes and environment. Fig. 16a shows that the subcooling circle and delayed structural change of GO–CNT encapsulated PCMs were avoided, indicating a timely and sufficient structural change of alkane in response to temperature change. This enables the encapsulated PCM to homogeneously dope, resulting in the “built-in” structure (Fig. 16b). The composites with dopant at 25 wt% maintain similar temperature-dependent electrical resistivity curves with the pristine GO joule heater. Notably, the composite maintains an around 5–10% enhancement in output temperature, either within PTR or the temperature lower or higher than the PTR (Fig. 16c).

### 3D printed functional composites

Additive fabrication technologies, better known as 3D printing, have witnessed an incredible development in recent years, owing to the versatile and low-cost method for rapid casting and prototyping. In this technology, the computational architecture is realised by solidifying liquid or pre-melted material *via* a straightforward layer-by-layer fabrication process. Many nanoscale functional materials have been introduced as nanofiller into printable resins and/or included in the pre-blending of materials,^199^ resulting in 3D printed composites exhibiting unique characteristics and capabilities, especially for controlled thermal properties. For example, 3D printing of composites with a relatively low amount of thermal conductive nanofillers, such as carbon nanotubes, graphene or metal nanoparticles, allows one to build objects with high thermal conductivity. The enhanced thermal conductivity accelerates the utilization of 3D printed composites in the heat sink and cooling system for heat management applications. Similar concepts can be applied to produce thermal insulation materials which have a variety of applications, such as reactionware for the chemistry.^200^

**Fig. 17:**
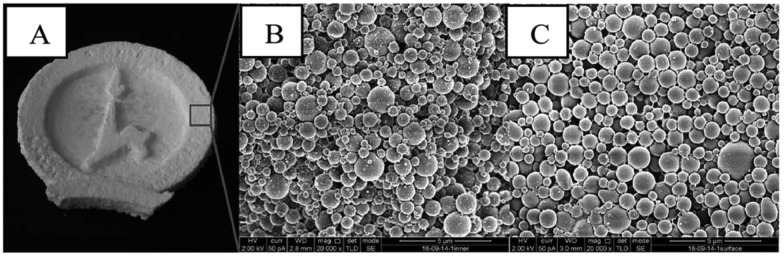
Images of printed emulsion with 60 wt% droplets phase through Digital Light Processing with an average diameter of 1.5 ± 0.03 mm (A) image of the device (B) SEM image of a cross-section (C) SEM image of the upper surface of the structure. Reproduced from ref. 202 with permission from the Royal Society of Chemistry, copyright 2015.

Commercial composite filaments for 3D printing systems are becoming widely available, produced by companies such as 3DXTech, Grafoid, Graphene 3D Lab, Haydale, *etc.* Relatively few technical problems are yet to be solved, such as temperature variations within a polymer matrix, aggregation at printing nozzle, inhomogeneity of resin, *etc.* Researchers have been shifting to print the functional nanoscale material itself without being used as the additive. We expect this emerging technique will facilitate the integration of nanoencapsulated PCM for advanced conceptual devices. Using the Digital Light Processing technique, Krajnc *et al.*^201^ printed a 3D hierarchical structure from a high internal phase emulsion (HIPE). The W/O emulsion contains 80 wt% droplet phase and photocurable continuous phase. Layer-by-layer assembly allows immobilization of droplets into free-standing complex 3D devices with excellent feature resolution. More recently, Magdassi *et al.*^202^ printed a monolithic porous structure from a 50–70 wt% O/W miniemulsion (Fig. 17).

Unlike the HIPE with high viscosity, the resin is composed of a UV polymerisable O/W dispersed phase which can feasibly be used in other printing techniques based on photo-polymerisation, for example photocurable inkjet printing.^203^ This method features simultaneous compartmentalization and device processing, allowing encapsulated materials to be directly made for applications. Besides light-based printing methods, direct ink writing of viscoelastic materials under ambient conditions offers a broad spectrum of printable materials for energy storage. Direct printing of nanoencapsulated PCMs is possible if the ink's viscosity, surface tension, shear yield stress, shear elastic and loss moduli can be properly tailored.

At the end, research on the applications of nanoencapsulated PCMs is still at an early stage, although some of researchers have approached the waste heat recovery, thermal management of electronic devices, energy conversion, intelligent building, “smart” air conditioning and thermal regulating fabric, *etc.* The main hurdle is to widen the choice of core and shell material through the optimal preparative process. Meanwhile, close-to-100% encapsulation efficiency, prolonged cyclability, tailorable thermal conductivity, more uniform particle size distribution and safety regards are the top criteria for the evaluation and commercialisation of nanoencapsulated PCM.

## 6. Conclusions & perspectives

Nanoencapsulation is one of the most promising solutions to increase the efficiency of PCMs, both organic and inorganic. It promotes high specific surface area, prevents exchange of encapsulated material with the environment, controls heat exchange across the capsule shell and initiates congruent melting/crystallisation due to the small core size. Energy nanocapsules can find new application fields in thermal energy storage, such as cascaded multi-temperature energy systems, additives to thermal paints or other building materials, *etc.*

However, current level of development of PCM encapsulation is mostly represented by macro and microencapsulation. The capsules consist of a single-layer polymer or oxide shell with only one functionality – prevention of material exchange with the environment. Future research should focus on the design of multifunctional composite PCM nanocapsules with enhanced thermal conductivity. Moreover, the effect of the nanoconfinement can change the crystallinity of PCMs in solid phase, which, in turn, can increase (or decrease) the melting enthalpy (heat uptake/release) in the confined nanovolume. This aspect of the nanoencapsulated PCMs is very poorly explored but has great potential to study nanosized effects in common salts or alkanes. We believe that there is an optimal size of the capsules, which can provide high efficiency of the storage of thermal energy. The nanoconfinement effect can overcome the losses of the latent heat energy caused be encapsulation. PCM-loaded macro and microcapsules do not have 100% encapsulation yield and, in spite of high stability, the heat capacity is usually lower than pure PCMs.

The widespread use of PCMs as energy storage materials can have vital consequences to aid humanity's drive for clean and renewable energy. Their various applications have highly advantageous effects, such as reduction in energy demand, reduced waste heat and improved efficiency for concentrated solar power plants. New methodology for capsule production needs to be developed further, such as complex emulsions, layer-by-layer assembly, microfluidics and industrial-scale sonication. These high-throughput manufacturing methods will lead to simple and wide-scale fabrication of PCM nanocapsules, reducing costs and increasing viability.

## Conflicts of interest

There are no conflicts to declare.

## References

[cit1] Xu B., Li P., Chan C. (2015). Appl. Energy.

[cit2] Lewis N. S., Nocera D. G. (2006). Proc. Natl. Acad. Sci. U. S. A..

[cit3] Walther G., Post E., Convey P., Menzel A., Parmesan C., Beebee T. J. C., Fromentin J., Hoegh-Guldberg O., Bairlein F. (2002). Nature.

[cit4] Thuiller W., Albert C., Araújo M. B., Berry P. M., Cabeza M., Guisan A., Hickler T., Midgley G. F., Paterson J., Schurr F. M., Sykes M. T., Zimmermann N. E. (2008). Perspect. Plant Ecol. Evol. Syst..

[cit5] Asif M., Muneer T. (2007). Renewable Sustainable Energy Rev..

[cit6] Siva Reddy V., Kaushik S. C., Ranjan K. R., Tyagi S. K. (2013). Renewable Sustainable Energy Rev..

[cit7] Tsao J., Lewis N., Crabtree G. (2006). US Dep. Energy.

[cit8] Grätzel M. (2001). Nature.

[cit9] Felix De Castro P., Ahmed A., Shchukin D. G. (2016). Chem. – Eur. J..

[cit10] Abhat A. (1983). Sol. Energy.

[cit11] Pardo P., Deydier A., Anxionnaz-Minvielle Z., Rougé S., Cabassud M., Cognet P. (2014). Renewable Sustainable Energy Rev..

[cit12] Prieto Cr., Cooper P., Fernandez A. I., Cabez L. F. (2016). Renewable Sustainable Energy Rev..

[cit13] Kenisarin M., Makhamov K. (2016). Renewable Sustainable Energy Rev..

[cit14] Soares N., Costa J. J., Gaspar A. R., Santos P. (2013). Energy Build..

[cit15] Akeiber H., Nejat P., Majid M. Z. A., Wahid M. A., Jomehzadeh F., Zeinali Famileh I., Kalautit J. K., Hughes B. R., Zaki S. A. (2016). Renewable Sustainable Energy Rev..

[cit16] Khateeb S. H., Amiruddin S., Farid M., Selman J. R., Al-Hallaj S. (2005). J. Power Sources.

[cit17] Moraga N. O., Xaman J. P., Araya R. H. (2016). Appl. Therm. Eng..

[cit18] Jiang G., Huang J., Fu Y., Cao M., Liu M. (2016). Appl. Therm. Eng..

[cit19] Ling Z., Zhang Z., Shi G., Fang X., Wang L., Gao X., Fang Y., Xu T., Wang S., Liu X. (2014). Renewable Sustainable Energy Rev..

[cit20] Browne M. C., Norton B., McCormack S. J. (2015). Renewable Sustainable Energy Rev..

[cit21] Smith C. J., Forster P. M., Crook R. (2014). Appl. Energy.

[cit22] Kim T. Y., Lee J. J., Rhee J. (2013). Aerosp. Sci. Technol..

[cit23] Liu X., Lou Y. (2015). Fibres Text. East. Eur..

[cit24] Sarier N., Onder E. (2012). Thermochim. Acta.

[cit25] Mondal S. (2008). Appl. Therm. Eng..

[cit26] Maruoka N., Mizuochi T., Purwanto H., Akiyama T. (2004). ISIJ Int..

[cit27] Nagano K., Ogawa K., Mochida T., Hayashi K., Ogoshi H. (2004). Appl. Therm. Eng..

[cit28] Johansson M. T., Söderström M. (2014). Energy Effic..

[cit29] Nomura T., Okinaka N., Akiyama T. (2010). ISIJ Int..

[cit30] Zheng Y., Barton J. L., Tuzla K., Chen J. C., Neti S., Oztekin A., Misiolek W. Z. (2015). Sol. Energy.

[cit31] Gimenez-Gavarrell P., Fereres S. (2017). Renewable Energy.

[cit32] Li J., Lu W., Luo Z., Zeng Y. (2017). Sol. Energy Mater. Sol. Cells.

[cit33] Karthikeyan M., Ramachandran T. (2014). Mater. Res. Innovations.

[cit34] Liu C., Rao Z., Zhao J., Huo Y., Li Y. (2015). Nano Energy.

[cit35] Chandel S. S., Agarwal T. (2017). Renewable Sustainable Energy Rev..

[cit36] Zalba B., Marin J. M., Cabeza L. F., Mehling H. (2003). Appl. Therm. Eng..

[cit37] Mohamed S. A., Al-Sulaiman F. A., Ibrahim N. I., Zahir M. H., Al-Ahmed A., Saidur R., Yılbaş B. S., Sahin A. Z. (2017). Renewable Sustainable Energy Rev..

[cit38] Naumann R., Emons H. H. (1989). J. Therm. Anal. Calorim..

[cit39] Stott P. W., Williams A. C., Barry B. W. (1998). J. Controlled Release.

[cit40] Myers P. D., Goswami D. Y. (2016). Appl. Therm. Eng..

[cit41] Kenisarin M., Mahkamov K. (2016). Sol. Energy Mater. Sol. Cells.

[cit42] Cabeza L. F., Illa J., Roca J., Badia F., Mehling H., Hiebler S., Ziegler F. (2001). Mater. Corros..

[cit43] Porisini F. C. (1988). Sol. Energy.

[cit44] Oró E., de Gracia A., Castell A., Farid M. M., Cabeza L. F. (2012). Appl. Energy.

[cit45] Ge H. S., Li H. Y., Mei S. F., Liu J. (2013). Renewable Sustainable Energy Rev..

[cit46] Ma K., Liu J. (2007). Frontiers of Energy Power Engineering in China.

[cit47] Ge H., Liu J. (2012). Front. Energy.

[cit48] Pereira da Cunha J., Eames P. (2016). Appl. Energy.

[cit49] Shukla A., Buddhi D., Sawhney R. L. (2008). Renewable Energy.

[cit50] Kenisarin M., Kenisarina K. (2012). Renewable Sustainable Energy Rev..

[cit51] Schossig P., Henning H. M., Gschwander S., Haussmann T. (2005). Sol. Energy Mater. Sol. Cells.

[cit52] Salunkhe P. B., Shembekar P. S. (2012). Renewable Sustainable Energy Rev..

[cit53] Shchukin D. G., Shchukina E. (2014). Curr. Opin. Pharmacol..

[cit54] Paul B. K., Mitra R. K. (2005). J. Colloid Interface Sci..

[cit55] Kundu K., Paul B. K. (2013). Colloids Surf., A.

[cit56] McClements D. J. (2012). Soft Matter.

[cit57] Peng L. C., Liu C. H., Kwan C. C., Huang K. F. (2010). Colloids Surf., A.

[cit58] Anton N., Benoit J. P., Saulnier P. (2008). J. Controlled Release.

[cit59] Rao J. P., Geckeler K. E. (2011). Prog. Polym. Sci..

[cit60] De La Rochebrochard S., Suptil J., Blais J. F., Naffrechoux E. (2012). Ultrason. Sonochem..

[cit61] Xiao R., Wei Z., Chen D., Weavers L. K. (2014). Environ. Sci. Technol..

[cit62] Teo B., Prescott S., Ashokkumar M., Grieser F. (2008). Ultrason. Sonochem..

[cit63] Bang J. H., Suslick K. S. (2010). Adv. Mater..

[cit64] Mason T. J. (2000). Ultrason. Sonochem..

[cit65] Suslick K. S., Price G. J. (1999). Annu. Rev. Mater. Sci..

[cit66] Suslick K. S., Flannigan D. J. (2008). Annu. Rev. Phys. Chem..

[cit67] Xu H., Zeiger B. W., Suslick K. S. (2013). Chem. Soc. Rev..

[cit68] Solans C., Izquierdo P., Nolla J., Azemar N., Garcia-Celma M. J. (2005). Curr. Opin. Colloid Interface Sci..

[cit69] Asakura Y., Nishida T., Matsuoka T., Koda S. (2008). Ultrason. Sonochem..

[cit70] Sathishkumar P., Mangalaraja R. V., Anandan S. (2016). Renewable Sustainable Energy Rev..

[cit71] Henglein A., Gutierrez M. (1990). J. Phys. Chem..

[cit72] Price G. J. (2003). Ultrason. Sonochem..

[cit73] Gibbs B. F., Kermasha S., Alli I., Mulligan C. (1999). Int. J. Food Sci. Nutr..

[cit74] Madene A., Jacquot M., Scher J., Desobry S. (2006). Int. J. Food Sci. Technol..

[cit75] Cheng L., He W., Gong H., Wang C., Chen Q., Cheng Z., Liu Z. (2013). Adv. Funct. Mater..

[cit76] Arnal P. M., Comotti M., Schüth F. (2006). Angew. Chem., Int. Ed..

[cit77] Bijlard A. C., Hansen A., Lieberwirth I., Landfester K., Taden A. (2016). Adv. Mater..

[cit78] Shchukin D. G., Zheludkevich M., Yasakau K., Lamaka S., Ferreira M. G. S., Möhwald H. (2006). Adv. Mater..

[cit79] Shchukin D. G., Möhwald H. (2007). Adv. Funct. Mater..

[cit80] Kafka A. P., McLeod B. J., Rades T., McDowell A. (2011). J. Controlled Release.

[cit81] Chitkara D., Kumar N. (2013). Pharm. Res..

[cit82] Vrignaud S., Anton N., Passirani C., Benoit J. P., Saulnier P. (2013). Drug Dev. Ind. Pharm..

[cit83] Panyam J., Labhasetwar V. (2003). Adv. Drug Delivery Rev..

[cit84] Mora-Huertas C. E., Fessi H., Elaissari A. (2010). Int. J. Pharm..

[cit85] Caruso F., Trau D., Moehwald H., Renneberg R. (2000). Langmuir.

[cit86] Cohen H., Levy R. J., Gao J., Fishbein I., Kousaev V., Sosnowski S., Slomkowski S., Golomb G. (2000). Gene Ther..

[cit87] Lomas H., Canton I., MacNeil S., Du J., Armes S. P., Ryan A. J., Lewis A. L., Battaglia G. (2007). Adv. Mater..

[cit88] Kang J., Rebek J. (1997). Nature.

[cit89] Chang T. M. S. (1964). Science.

[cit90] Couvreur P., Speiser P., Tulkens P., Roland M., Trouet A. (1977). FEBS Lett..

[cit91] Ballenger J., Post R. (1980). Am. J. Psychiatry.

[cit92] Gupta A. K., Gupta M. (2005). Biomaterials.

[cit93] Moghimi S. M. S. M., Hunter A. C. A. C., Murray J. C. J. C. (2001). Pharmacol. Rev..

[cit94] Rytkönen J., Miettinen R., Kaasalainen M., Lehto V. P., Salonen J., Närvänen A. (2012). J. Nanomater..

[cit95] Zhang G. H., Bon S. A. F., Zhao C. Y. (2012). Sol. Energy.

[cit96] Jamekhorshid A., Sadrameli S. M., Farid M. (2014). Renewable Sustainable Energy Rev..

[cit97] Soottitantawat A., Yoshii H., Furuta T., Ohkawara M., Linko P. (2003). J. Food Sci..

[cit98] Gharsallaoui A., Roudaut G., Chambin O., Voilley A., Saurel R. (2007). Food Res. Int..

[cit99] Luo Y. W., Zhou X. D. (2004). J. Polym. Sci., Part A: Polym. Chem..

[cit100] Tiarks F., Landfester K., Antonietti M. (2001). Langmuir.

[cit101] Paiphansiri U., Tangboriboonrat P., Landfester K. (2006). Macromol. Biosci..

[cit102] Utama R. H., Stenzel M. H., Zetterlund P. B. (2013). Macromolecules.

[cit103] Gröhn F., Bauer B. J., Amis E. J. (2001). Macromolecules.

[cit104] Decher G. (1997). Science.

[cit105] Sukhorukov G. B., Möhwald H. (2008). Trends Biotechnol..

[cit106] Shchukin D. G., Ustinovich E., Sukhorukov G. B., Möhwald H., Sviridov D. V. (2005). Adv. Mater..

[cit107] Zhang X., Chen H., Zhang H. (2007). Chem. Commun..

[cit108] Stockton W. B., Rubner M. F. (1997). Macromolecules.

[cit109] Fang M. M., Kaschak D. M., Sutorik A. C., Mallouk T. E. (1997). J. Am. Chem. Soc..

[cit110] Johnston A. P. R., Read E. S., Caruso F. (2005). Nano Lett..

[cit111] Wang Z. P., Feng Z. Q., Gao C. Y. (2008). Chem. Mater..

[cit112] Lojou T., Bianco P. (2004). Langmuir.

[cit113] Lvov Y., Ariga K., Ichinose I., Kunitake T. (1995). Chem. Commun..

[cit114] Sukhorukov G., Fery A., Möhwald H. (2005). Prog. Polym. Sci..

[cit115] Moya S., Sukhorukov G. B., Auch M., Donath E., Mohwald H. (1999). J. Colloid Interface Sci..

[cit116] Shchukina E. M., Shchukin D. G. (2011). Adv. Drug Delivery Rev..

[cit117] Grigoriev D. O., Bukreeva T., Möhwald H., Shchukin D. G. (2008). Langmuir.

[cit118] Oya T., Nomura T., Tsubota M., Okinaka N., Akiyama T. (2013). Appl. Therm. Eng..

[cit119] Qi G. Q., Yang J., Bao R. Y., Liu Z. Y., Yang W., Xie B. H., Yang M. B. (2015). Carbon.

[cit120] Haase M. F., Grigoriev D., Möhwald H., Tiersch B., Shchukin D. G. (2011). Langmuir.

[cit121] Bedard M., Munoz-Javier A., Mueller R., Del Pino P., Feri A., Parak W. J., Skirtach A., Sukurukov G. B. (2009). Soft Matter.

[cit122] Asua J. M. (2014). Prog. Polym. Sci..

[cit123] Asua J. M. (2002). Prog. Polym. Sci..

[cit124] Landfester K., Eisenblätter J., Rothe R. (2004). J. Coat. Technol. Res..

[cit125] Yin D. Z., Ma L., Liu J. J., Zhang Q. Y. (2014). Energy.

[cit126] Yang J., Keller M. W., Moore J. S., White S. R., Sottos N. R. (2008). Macromolecules.

[cit127] Huang X., Voit B. (2013). Polym. Chem..

[cit128] Zarzar L. D., Sresht V., Sletten E. M., Kaliw J. A., Blankschtein D., Swager T. M. (2015). Nature.

[cit129] Theunissen P.-H., Buchlin J.-M. (1983). Sol. Energy.

[cit130] Felix De Castro P., Shchukin D. G. (2015). Chem. – Eur. J..

[cit131] Zhang H., Wang X. (2009). Sol. Energy Mater. Sol. Cells.

[cit132] Barlak S., Sara O. N., Karaipekli A., Yapici S. (2016). Nanoscale Microscale Thermophys. Eng..

[cit133] Alkan C., Sari A., Karaipekli A. (2011). Energy Convers. Manage..

[cit134] Alkan C., Sari A., Biglin C. (2014). Appl. Energy.

[cit135] Yi Q., Sukhorukov G. B., Ma J., Yang X., Gu Z. (2015). Int. J. Polym. Sci..

[cit136] Zhang X. X., Fan Y. F., Tao X. M., Yick K. L. (2004). Mater. Chem. Phys..

[cit137] Fan Y. F., Zhang X. X., Wang X. C., Li J., Zhu Q. B. (2004). Thermochim. Acta.

[cit138] Konuklu Y., Unal M., Paksoy H. O. (2014). Sol. Energy Mater. Sol. Cells.

[cit139] Chen Z. H., Zeng X. R., Zhang G. (2012). Appl. Energy.

[cit140] Sari A., Alkan C., Karaipekli A. (2010). Appl. Energy.

[cit141] Yang R., Xu H., Zhang Y. (2003). Sol. Energy Mater. Sol. Cells.

[cit142] Fang Y., Liu X., Liang X., Liu H., Gao X., Zhang Z. (2014). Appl. Energy.

[cit143] Tumirah K., Hussein M. Z., Zulkarnain Z., Rafeadah R. (2014). Energy.

[cit144] Fuensanta M., Paibhansiri U., Romero-Sanches M. D., Guillem C., Lopez-Buendia A. M., Landfester K. (2013). Thermochim. Acta.

[cit145] Latibari S. T., Mehrali M., Mehrali M., Meurah T., Malia T. M. I., Metselaar H. S. C. (2013). Energy.

[cit146] Wang Y., Zhang Y., Xia T., Zhao W., Yang W. (2014). Sol. Energy Mater. Sol. Cells.

[cit147] Fang G., Chen Z., Li H. (2010). Chem. Eng. J..

[cit148] Zhang H., Wang X., Wu D. (2010). J. Colloid Interface Sci..

[cit149] Geng L. X., Wang S. F., Wang T. Y., Luo R. L. (2016). Energy Fuels.

[cit150] Fu W., Liang X., Xie H., Wang S., Gao X., Zhang Z., Fang Y. (2017). Energy Build..

[cit151] Shi J., Wu X., Fu X., Sun R. (2015). Thermochim. Acta.

[cit152] Zhu Y., Liang S., Wang H., Zhang K., Jia X., Tian C., Zhou Y., Wang J. (2016). Energy Convers. Manage..

[cit153] Zhu Y., Liang S., Chen K., Gao X., Chang P., Tian C., Wang J., Huang Y. (2015). Energy Convers. Manage..

[cit154] Rao Z., Wang S., Peng F. (2012). Appl. Energy.

[cit155] Demirbag S., Aksoy S. A. (2016). Fibers Polym..

[cit156] Demirbag S., Aksoy S. A. (2013). J. Text. Eng..

[cit157] Zheng Z., Jin J., Xu G.-K., Zou J., Wais U., Beckett A., Heil T., Higgins S., Guan L., Wang Y., Shchukin D. (2016). ACS Nano.

[cit158] Zheng Z., Chang Z., Xu G. K., McBride F., Ho A., Zhuola Z., Michailidis M., Raval R., Akhtar R., Shchukin D. G. (2017). ACS Nano.

[cit159] Fan L. W., Zhu Z. Q., Liu M. J., Xu C. L., Zeng Y., Lu H., Yu Z. T. (2016). J. Heat Transfer.

[cit160] Phadungphatthanakoon S., Pumpradub S., Wanichwecharungruang S. (2011). ACS Appl. Mater. Interfaces.

[cit161] Kalashnikova I., Bizot H., Kathala B., Capron I. (2011). Langmuir.

[cit162] Svagan A. J., Musyanovich A., Cappl M., Bernhardt M., Glasser G., Wohnhaas C., Berglund L. A., Risbo J., Landfester K. (2014). Biomacromolecules.

[cit163] Li Y., Yu S., Chen P., Rojas R., Hajan A., Berglund L. (2017). Nano Energy.

[cit164] Huang J., Wang T. Y., Zhu P. P., Xiao J. B. (2013). Thermochim. Acta.

[cit165] Platte D., Helbig U., Houbertz R., Sextl G. (2013). Macromol. Mater. Eng..

[cit166] Milian Y. E., Gutierrez A., Gragada M., Ushak S. (2017). Renewable Sustainable Energy Rev..

[cit167] Sarier N., Onder E. (2007). Thermochim. Acta.

[cit168] Sarier N., Onder E. (2007). Thermochim. Acta.

[cit169] Salaün F., Devaux E., Bourbigot S., Rumeau P. (2008). Carbohydr. Polym..

[cit170] Liu C., Wang C., Li Y., Rao Z. (2017). RSC Adv..

[cit171] Graham M., Shchukina E., Felix De Castro P., Shchukin D. G. (2016). J. Mater. Chem. A.

[cit172] El-Sebaii A. A., A-Heniti S., Al-Agel F., Al-ghamidi A. A., Al-Marzouki F. (2001). Energy Convers. Manage..

[cit173] Graham M., Cola-Clemente J., Shchukina E., Shchukin D. G. (2017). J. Mater. Chem..

[cit174] Wang T., Huang J., Zhu P., Xiao J. (2013). Colloid Polym. Sci..

[cit175] Schoth A., Landfester K., Muñoz-Espí R. (2015). Langmuir.

[cit176] Hassabo A. G., Mohamed A. L., Wang H., Popescu C., Moller M. (2015). Inorg. Chem.: Indian J..

[cit177] Zhang J., Wang S. S., Zhang S. D., Tao Q. H., Pan L., Wang Z. Y., Zhang Z. P., Lei Y., Yang S. K., Zhao H. P. (2011). J. Phys. Chem. C.

[cit178] Zhang G., Li J., Chen Y., Xiang H., Ma B., Xu Z., Ma X. (2014). Sol. Energy Mater. Sol. Cells.

[cit179] Karthikeyan M., Ramachandran T., Sundaram O. L. S. (2014). J. Ind. Text..

[cit180] Karthikeyan M., Ramachandran T., Shanmugasundaram O. L. (2014). J. Text. Inst..

[cit181] Sarı A., Alkan C., Biçer A., Altuntaş A., Bilgin C. (2014). Energy Convers. Manage..

[cit182] Ray P. C., Yu H. T., Fu P. P. (2009). J. Environ. Sci. Health, Part C: Environ. Carcinog. Ecotoxicol. Rev..

[cit183] Sharifi S., Behzadi S., Laurent S., Forrest M. L., Stroeve P., Mahmoudi M. (2012). Chem. Soc. Rev..

[cit184] Goel M., Roy S. K., Sengupta S. (1994). Int. J. Heat Mass Transfer.

[cit185] Khodadadi J. M., Hosseinizadeh S. F. (2007). Int. Commun. Heat Mass Transfer.

[cit186] Wang Y., Chen Z. Q., Ling X. (2016). Appl. Therm. Eng..

[cit187] Dumas J. P., Krichi M., Strub M., Zeraouli Y. (1994). Int. J. Heat Mass Transfer.

[cit188] Hu X., Zhang Y. (2002). Int. J. Heat Mass Transfer.

[cit189] Han Z. H., Cao F. Y., Yang B. (2008). Appl. Phys. Lett..

[cit190] Liu C. Z., Rao Z. H., Zhao J. T., Huo Y. T., Li Y. M. (2015). Nano Energy.

[cit191] Rehman M. M. U., Qu Z. G., Fu R. P., Xu H. T. (2017). Int. J. Heat Mass Transfer.

[cit192] Wu W., Bostanci H., Chow L. C., Hong Y., Wang C. M., Su M., Kizito J. P. (2013). Int. J. Heat Mass Transfer.

[cit193] Liu M., Bruno F., Saman W. (2011). Sol. Energy.

[cit194] Youssef Z., Delahaye A., Huang L., Trinquet F., Fournaison L., Pollerberg C., Doetsch C. (2013). Energy Convers. Manage..

[cit195] Seyf H. R., Zhou Z., Ma H. B., Zhang Y. (2013). Int. J. Heat Mass Transfer.

[cit196] Michels H., Pitz-Paal R. (2007). Sol. Energy.

[cit197] Rehman M. M. U., Qu Z. G., Fu R. P. (2016). J. Therm. Sci..

[cit198] Bae J. J., Lim S. C., Han G. H., Jo Y. W., Doung D. L., Kim E. S., Chae S. J., Huy T. Q., Luan N. V., Lee Y. H. (2012). Adv. Funct. Mater..

[cit199] Kalsoom U., Nesterenko P. N., Paull B. (2016). RSC Adv..

[cit200] Symes M. D., Kitson P. J., Yan J., Richmond C. J., Cooper G. J. T., Bowman R. W., Vilbrandt T., Cronin L. (2012). Nat. Chem..

[cit201] Sušec M., Ligon S. C., Stampfl J., Liska R., Krajnc P. (2013). Macromol. Rapid Commun..

[cit202] Cooperstein I., Layani M., Magdassi S. (2015). J. Mater. Chem. C.

[cit203] Truby R. L., Lewis J. A. (2016). Nature.

